# Genome-Wide Association Study Reveals Genetic Architecture of Eating Behavior in Pigs and Its Implications for Humans Obesity by Comparative Mapping

**DOI:** 10.1371/journal.pone.0071509

**Published:** 2013-08-19

**Authors:** Duy Ngoc Do, Anders Bjerring Strathe, Tage Ostersen, Just Jensen, Thomas Mark, Haja N Kadarmideen

**Affiliations:** 1 Department of Veterinary Clinical and Animal Sciences, Faculty of Health and Medical Sciences, University of Copenhagen, Frederiksberg, Denmark; 2 Danish Agriculture and Food Council, Pig Research Centre, Copenhagen, Denmark; 3 Aarhus University, Department of Molecular Biology and Genetics, Tjele, Denmark; Democritus University of Thrace, Greece

## Abstract

This study was aimed at identifying genomic regions controlling feeding behavior in Danish Duroc boars and its potential implications for eating behavior in humans. Data regarding individual daily feed intake (DFI), total daily time spent in feeder (TPD), number of daily visits to feeder (NVD), average duration of each visit (TPV), mean feed intake per visit (FPV) and mean feed intake rate (FR) were available for 1130 boars. All boars were genotyped using the Illumina Porcine SNP60 BeadChip. The association analyses were performed using the GenABEL package in the R program. Sixteen SNPs were found to have moderate genome-wide significance (p<5E-05) and 76 SNPs had suggestive (p<5E-04) association with feeding behavior traits. *MSI2* gene on chromosome (SSC) 14 was very strongly associated with NVD. Thirty-six SNPs were located in genome regions where QTLs have previously been reported for behavior and/or feed intake traits in pigs. The regions: 64–65 Mb on SSC 1, 124–130 Mb on SSC 8, 63–68 Mb on SSC 11, 32–39 Mb and 59–60 Mb on SSC 12 harbored several signifcant SNPs. Synapse genes (*GABRR2, PPP1R9B, SYT1, GABRR1, CADPS2, DLGAP2* and *GOPC)*, dephosphorylation genes (*PPM1E, DAPP1, PTPN18, PTPRZ1, PTPN4, MTMR4* and *RNGTT*) and positive regulation of peptide secretion genes (*GHRH, NNAT* and *TCF7L2*) were highly significantly associated with feeding behavior traits. This is the first GWAS to identify genetic variants and biological mechanisms for eating behavior in pigs and these results are important for genetic improvement of pig feed efficiency. We have also conducted pig-human comparative gene mapping to reveal key genomic regions and/or genes on the human genome that may influence eating behavior in human beings and consequently affect the development of obesity and metabolic syndrome. This is the first translational genomics study of its kind to report potential candidate genes for eating behavior in humans.

## Introduction

Feed represents a large proportion of the variable costs of breeding. Therefore, selection for reducing feed intake is a very important goal in breeding programs, at least in Danish pig breeds. Genetic improvement in feed efficiency was historically achieved as a correlated genetic change resulting from selection for growth rate and carcass lean content for animals tested in groups, where individual feed intake was too expensive to be measured on a large number of pigs. In recent years, the study of feed intake and behavior in pigs has been greatly facilitated by development of computerized systems that record the feed intake and related measures of individual animals within a group each time they enter the feeder. Several studies have shown low to moderate and positive genetic correlation between feeding behavior traits and daily feed intake. For instance, DFI had a positive genetic correlation with NVD (r = 0.27) [1]. Labroue *et al.* [2] found FPV had positive genetic correlation to average daily gain, meaning that animals that eat more per visit tend to grow faster. These genetic associations underline the fact that genetic improvement of feed efficiency is also dependant upon genetic changes (improvement) in eating behavior of pigs. Furthermore, genomic control and gene pathways involved in eating or feeding behavior and its association to weight gain in pigs may translate to human eating behavior and obesity, because the pig is an excellent animal model genetically and physiologically very similar to humans [3]. Feeding behavior has been reported to be highly related to social interaction of pigs and the number of pigs competing for access to the same feeder. Nielsen *et al*. [4] found that pigs with more frequent visits to the feeder were found to be positively correlated with less competition. Knowledge of molecular mechanisms of feeding behavior might help to improve our understanding of behavioral problems that are common in many fields of animal production (e.g. aggression, stress, pain). Quantitative trait loci (QTL) mapping is the first step to detect chromosomal regions affecting complex traits. Approximately 70 QTLs have previously been detected for feeding, drinking and socializing behaviors on 15 different pig chromosomes to date (http://www.animalgenome.org/cgi-bin/QTLdb/SS/index). However, QTLs are often mapped by linkage analysis to a large interval of 20 centimorgans (cM) or more that may contain several hundreds of genetic variants, not ideal for accurate mapping of potential causal variants [5]–[7]. Genome-wide association studies (GWAS) that survey most of the genome using dense genomic markers have been developed and applied widely in the analysis of complex traits in animals [8] and humans [9]. GWAS take advantage of a large numbers of SNP markers in population-wide linkage disequilibrium with very small (QTL) regions potentially harboring candidate loci for the complex traits. Although some studies have identified QTLs for pig feeding behavior traits, this is is the first GWAS conducted to identify genetic variants and biological mechanisms for eating behavior in pigs.

The obesity epidemic has become one of the most important public health problems [10] and many of the common genetic variants for the risk of obesity, metabolic syndrome and related complications are associated with specific eating behaviors in human [11]. A number of studies have shown that pigs are an excellent model for human obesity and metalolic syndrome [3]. Eating behavior in humans (e.g. compulsive or comfort eating) can also be studied using the pig model, because eating behavior is closely related to development of obesity and metabolic syndrome. One of the objectives of this study was to conduct comparative pig-human genome mapping to identify potential candidate genes that may affect the way humans eat and develop obesity and related metabolic syndrome.

## Materials and Methods

### Recording of feeding behavior traits

A total of 7388 Duroc pigs had phenotypic records from the period of 2008–2011 and 1909 boars had 60 k SNP genotype records. The selection of boars to be genotyped and sent to the test station (i.e. phenotyping) was primarily based on their aggregate breeding value, but feeding behavior is not directly part of the breeding goal of Danish pig breeds and some genotyped boars have no recorded phenotype. For GWAS, anmials had to have both phenotypic and genotypic information; 1130 boars that had both genotypic and phenotypic records for feeding behavior traits were used in the study. Summary statistics of phenotypes are shown in Table 1. Data were recorded at the central Danish pig test station (Bøgildgård) during a period of four years (2008–2011) and the data were supplied by the Pig Research Centre of the Danish Agriculture and Food Council. The details of management and data records were described in Duy *et al*. [1]. In summary, boars were put into pens of approx. 11 boars. Each pen had one ACEMO automatic dry feeding station and the boars were fed *ad libitum* from 30 kg to approximately 100 kg live weight with the same feed composition. The time, duration and feed consumption was recorded for each individual visit. Average daily feed intake (DFI) was derived from the total amount of recorded feed intake divided by the number of corresponding days at the feeder. The following feeding behavior traits were defined and calculated for each boar: DFI: total daily feed intake (kg/d), TPD: total time spent at feeder per day (minute), NVD: number of visits to the feeder per day, TPV: average duration of each visit ( =  TPD/NVD), FPV: mean feed intake per visit (kg) and FR: mean feed intake rate (g/minute) ( = DFI/TPD) [1].

**Table 1:** Descriptive statistics (mean ± SD), reliability of Evaluated Breeding Value (EBV) for measured traits in Duroc boars.

Abbreviation	Trait	Units	Mean ± SD	Reliability of EBV
DFI	Total daily feed intake	kg	2.34±0.40	0.48±0.03
NVD	Number of visits to feeder per day	count	10.06±5.21	0.48±0.04
TPD	Total time spent at feeder per day	min	78.35±13.51	0.54±0.05
TPV	Time spent to eat per visit	min	8.18±3.62	0.46±0.02
FR	Mean feed intake rate	g/min	30.54±0.67	0.55±0.02
FPV	Mean feed intake per visit	kg	0.027±0.01	0.52±0.03

### Generating dependent variable for GWAS

The estimated breeding values (EBVs) for DFI and feeding behaviors were calculated by single-trait animal model with fixed effect of herd-year-season, random effect of pen and a random additive genetic effect, as in Duy *et al*. [1]. The phenotype used for association analysis was deregressed estimated breeding values (EBVs). The details of the estimation of deregressed EBVs are given by Ostersen *et al*. [12] following the deregression procedure of Garrick *et al*. [13]. Briefly, the deregression adjusts for ancestral information, such that the deregressed EBV only contains their own and the descendant's information on each animal to avoid regressing information in both the generation of the dependent variable and the subsequent GWAS.

### Genotyping and data validation

The details of the genotyping method have been described previously [12], [14]. In summary, genomic DNA was isolated from all specimens by treatment with proteinase K followed by sodium chloride precipitation and SNPs were genotyped on the PorcineSNP60 Illumina iSelect BeadChip. The inclusion criteria for genomic data was a call rate per animal of 0.95. The inclusion criteria for SNP markers were a call rate of 0.95, Hardy Weinberg equilibrium test with p<0.0001 and minor allele frequency >0.05.

### Statistical models for GWAS

The relationship matrix used by the “polygenic” linear mixed effects model was generated by the *ibs()* function of GenABEL which uses identity by state (IBS) genotype sharing to determine the realised pairwise kinship coefficient. Then a genome-wide association analysis was performed using a score test, a family-based association test, implemented in the *mmscore()* function of *R/GenABEL* [15]. The full model: *y*  =  X*b* + W*p* + Z*a* + *e (1)* is implemented in two steps in GenABEL. In the equation (*1*), ***y*** is the vector deregressed EBVs for a given trait, X is an incidence matrix for fixed non-genetic effects *b* (herd–week section and pen), *W* is a vector with genotypic indicators (−1, 0, or 1) associating records to the marker effect, *p* is a scalar of the associated additive effect of the SNP, **Z** is an incidence matrix relating phenotypes to the corresponding random polygenic effect, *a* is a vector of the random polygenic effect with the normal distribution *a* ∼ N(0, 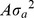), where **A** is the additive relationship matrix and 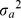 is the polygenic variance, and **e** is a vector of random environmental deviates with the normal distribution 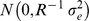, where 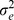 is the error variance and **R** is the diagonal matrix containing weights of the deregressed estimated breeding values. Instead of fitting this full mixed model everytime a single SNP is fitted, the reduced model without the term *Wp* (SNP effect) is fitted only once and all fixed, polygenic and residual components are estimated using the REML approach. In the second step, with the estimated heritability estimate and kinship coefficients for each pair of relatives, the correlation between phenotypic records of relatives are adjusted and approximate IID (identical and independantly distributed) phenotypes with normality are obtained.

This *mmscore* test for family-based association is then conducted on the adjusted phenotype from the second step which takes into account pedigree structure and allows unbiased estimations of SNP allelic effect when relatedness is present between examinees [16]. Multidimensional scaling plot of kindship distance based on IBS was used to check outliers and possible population stratification. The influence of population stratification after genomic control was also assessed in a quantile-quantile (q-q) plot by examining the distribution of test statistics generated from association tests and the deviation from the null hypothesis of no SNP association with the trait was assessed [17]. The inflation factors before and after genomic control were 1.88 and 1.01, 2.16 and 1.04, 1.69 and 1.05, 2.14 and 1, 1.86 and 1.01 and 1.87 and 1.03 for DFI, TPD, NVD, TPV, FPV and FR, respectively. The genome-wide significance association at 5% significance level after Bonferroni multiple testing correction was p = 1.56E-06. However, the Bonferroni correction may result in a too stringent or very conservative threshold [18] and hence result in many false negative results, as this method assumes markers are independent. This is not the case in reality due to linkage disequilibrium (LD) between markers. Therefore, to avoid many false negative results caused by Bonferroni correction, the loci with p<5E-05 were considered as moderately genome-wide significant and loci with p<5E-04 were considered to be suggestively genome-wide significant. Both types of signifcant SNPs were included in downstream bioinformatics analysis. Linkage disequilibrium (LD) between SNPs in the chromosomal regions where multiple candidate SNPs were located was quantified as D' on all the animals of the GWAS using Haploview V4.2 [19] and the LD block was defined by the criteria in [20]. Frequency of defined haplotypes and their contribution to phenotypic variances of related traits was calculated using the PLINK software [21].

### Bioinformatics analyses

SNP positions were updated according to the newest release from Ensembl (Sscrofa10.2 genome version). Comparative mapping was performed by annotating significant SNP position to previously mapped QTL in pigs using the pig QTL database: http://animalgenome.org/cgi-bin/QTLdb/index [22] (assessed on 3rd, Feb, 2013). We also attempted to perform comparative mapping of chromosomal regions containing high numbers of *tag* (significant and suggestive) SNPs with human genomic map using RH map and comparative maps provided by Mayer *et al*., [23] in the QTL database [22]. Identification of the closest genes to *tag* SNPs was obtained using Ensembl annotation of Sscrofa10.2 genome version (http:// ensembl.org/Sus_scrofa/Info/Index). The positional candidate genes within 1 Mb bin size on either side of top SNPs peak were scanned using the function *GetNeighGenes()* in the NCBI2R package at http://cran.r-project.org/web/packages/NCBI2R/index.html using the R program [24]. Investigation of functional categories in nearby genes was performed using the Database for Annotation, Visualization and Integrated Discovery (DAVID) at http://david.abcc.ncifcrf.gov/ [25]. Human genes were used as background in annotation analysis, because many nearby genes have not been characterized in pigs and because translational gene aspects are of high interest.

## Results

### Quality control, populations stratification assessments and phenotypic variation explained by markers

Following quality control of SNP data, 23795 markers were excluded as having a low (<5%) minor allele frequency, 1836 markers were excluded because of low (<95%) call rate and 3463 markers were excluded because they were not in HWE (p<0.001). A final set of 33945 SNPs and 1130 pigs was retained for GWAS. The number of markers on each chromosome and average distances between two markers after quality control are given in Table S1. Multidimensional scaling plot of IBS distances showed no outliers in populations (Figure S1). Total variance of all SNP markers explained 33, 42, 25, 38, 36 and 37% of the phenotypic variance (of the dependent variable, dEBVs) for DFI, TPD, NVD, TPV, FPV and FR, respectively.

### Genome-wide association analysis and functional categories of nearby genes

**Figure 1:**
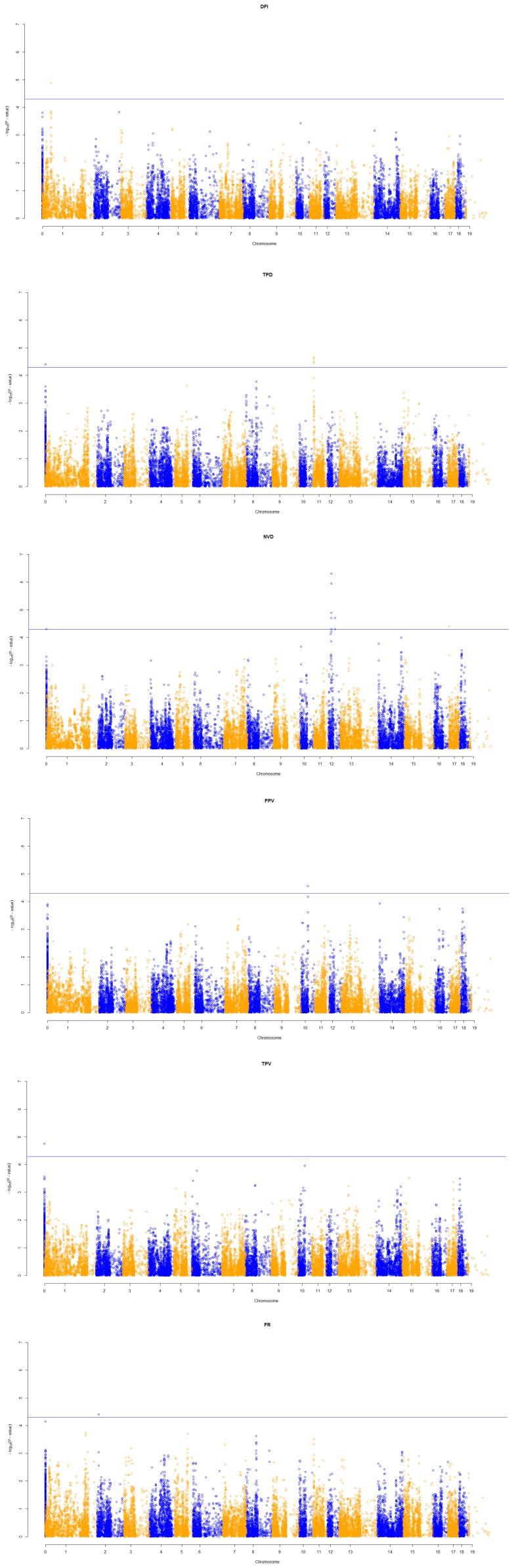
Manhattan plot showing association with feeding behavior traits for all the SNPs. The horizontal line indicates genome-wide significant threshold. On vertical, Manhattan plot for total daily feed intake (DFI), total time spent at feeder per day (TPD), number of visits to the feeder per day (NVD), time spent to eat per visit (TPV), mean feed intake per visit (FPV), and mean feed intake rate (FR), respectively. Chromosome 19 stands for X chromosome. Chromosome 0 stands for unmapped SNPs.

**Figure 2:**
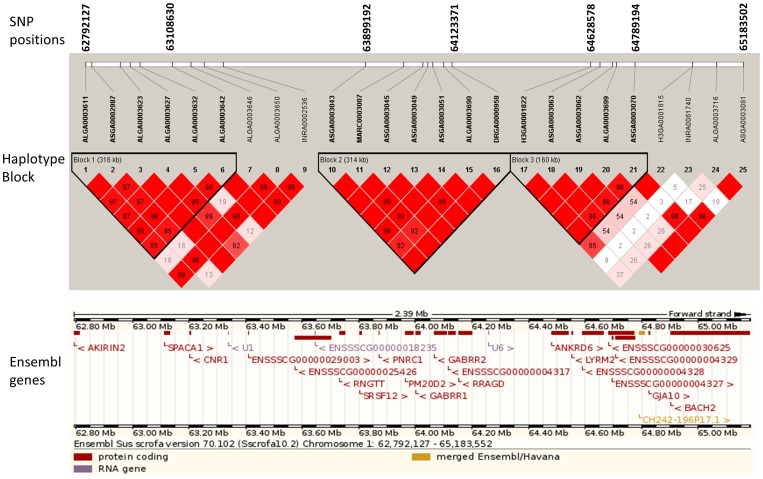
Linkage disequilibrium (LD) pattern and Ensemble genes on region from 62–65 Mb on pig chromosome 1. LD blocks are marked with triangles. Values in boxes are LD (*r^2^*) between SNP pairs and the boxes are colored according to the standard Haploview color scheme: LOD >2 and D'  = 1, red; LOD >2 and D'<1, shades of pink/red; LOD<2 and D'  = 1, blue; LOD<2 and D'<1, white (LOD is the log of the likelihood odds ratio, a measure of confidence in the value of D').

**Figure 3:**
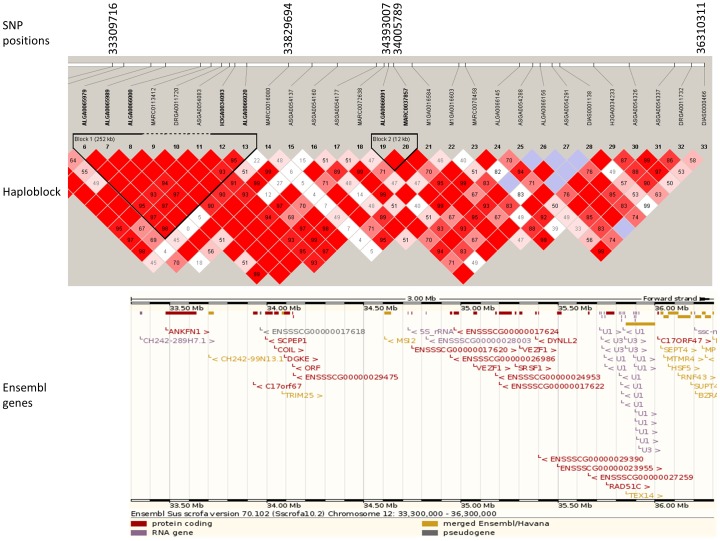
Linkage disequilibrium (LD) pattern and Ensemble genes on region from 33.5–35.5 Mb on pig chromosome 12. LD blocks are marked with triangles. Values in boxes are LD (*r^2^*) between SNP pairs and the boxes are colored according to the standard Haploview color scheme: LOD >2 and D'  = 1, red; LOD >2 and D'<1, shades of pink/red; LOD<2 and D'  = 1, blue; LOD<2 and D'<1, white (LOD is the log of the likelihood odds ratio, a measure of confidence in the value of D').

Among 92 significant SNPs, 16 were found to have moderate genome-wide significance (Table 2) and 76 were found to have suggestive (Table S2) associations with feeding behavior traits. Number of significant and suggestive loci associated with DFI, TPD, NVD, TPV, FPV and FR were 1 and 10, 6 and 11, 6 and 16, 1 and 10, 1 and 19 and 1 and 10, respectively. While associated SNP with DFI, TPD and NVD were located on SSC 1, 11 and 12, the associated SNP with other traits were distributed around different chromosomes. Eleven SNPs were in unassembled scaffolds of the Sscrofa10.2 genome version. The locus DRGA00169471 on SSC 18 was found associated with both TPF and FPV. Nineteen of 92 loci were found in the intronic regions of known genes. The chromosomes and exact positions based on Sus scrofa Genebuild 10.2 (SSC10.2 build) as well as their nearest genes for SNPs were listed in Table 2. Quantitile-quantitle plots of observed and expected p values and Manhattan plots of GWAS of all traits after genomic control are shown in Figure S2 and Figure 1, respectively. Three haplotype blocks were detected in genomic regions affecting DFI on SSC1 (Figure 2). The major haplotypes with occurrence frequency is shown in Table 3. Two haplotype blocks were detected in genomic regions influencing NVD on SSC 12 (Figure 3) and their frequency and contribution to variances of the trait are shown in Table S4.

**Table 2:** Significant SNP associated to studied eating behavioral traits, their positions and nearest genes and distance from SNPs to corresponding genes.

Trait^1^	SNP^2^	SSC^3^	Position	Ensembl Gene ID	Gene	Distances^4^ (bp)	P_GC_ 5	P_raw_ 6
DFI	ALGA0003690	1	64094344	*ENSSSCG00000024249*	*GABRR2*	intron	1.35E-05	5.70E-07
FPV	MARC00914141	10	38641420	*ENSSSCG00000023807*	*ACO1*	−446568	3.18E-05	4.19E-08
FR	H3GA0006163	2	18828505	*ENSSSCG00000013277*	*TP53I11*	97726	5.00E-05	1.79E-05
NVD	M1GA0016584	12	34552177	*ENSSSCG00000017619*	*MSI2*	57256	9.65E-07	2.34E-09
NVD	ASGA0054177	12	34360905	*ENSSSCG00000017619*	*MSI2*	248528	2.19E-06	5.46E-09
NVD	ASGA0054288	12	34781411	*ENSSSCG00000017619*	*MSI2*	−33633	2.27E-05	2.66E-08
NVD	MARC0070458	12	34719298	*ENSSSCG00000017619*	*MSI2*	28480	2.27E-05	2.66E-08
NVD	ALGA0066091	12	34393007	*ENSSSCG00000017619*	*MSI2*	216436	3.49E-05	4.30E-08
NVD	MARC0072638	12	34381325	*ENSSSCG00000017619*	*MSI2*	366453	3.49E-05	4.30E-08
NVD	MARC0097496	12	39543788	*ENSSSCG00000017682*	*MYO19*	10919	3.51E-05	4.40E-08
TPD	MARC0085057	5	101511939	*ENSSSCG00000000933*	*ALX1*	−13827	3.97E-05	4.40E-04
TPD	ASGA0049606	11	8523653	*ENSSSCG00000009337*		24003	2.27E-05	9.48E-09
TPD	ASGA0049612	11	8505201	*ENSSSCG00000009338*		intron	2.27E-05	9.48E-09
TPD	ALGA0060596	11	7421327	*ENSSSCG00000009332*	*TEX26*	intron	2.44E-05	2.20E-09
TPD	ASGA0049581	11	6392619	*ENSSSCG00000000615*		8005	3.19E-05	1.40E-09
TPD	ALGA0060626	11	6443449	*ENSSSCG00000000615*		−42825	3.59E-05	1.38E-09
TPV	M1GA00245241	12	59746968	*ENSSSCG00000028465*	*ELAC2*	349815	1.79E-05	6.40E-05

1 : DFI: total daily feed intake, FPV: mean feed intake per visit, FR: mean feed intake rate, NVD: number of visits to the feeder per day, TPD: total time spent at feeder per day, TPV: time spent to eat per visit.

2 : SNP names according to Illumina- Porcine beadchips.

3 : Pig chromosomes.

4 : Distance from SNPs to starting point of genes.

5 : P_GC_: GWAS p-value after genomic control.

6 : P_raw_: GWAS p-value before genomic control.

**Table 3:** Haplotypes and their frequencies in the candidate region for total daily feed intake on chromosome 1.

Locus	Haplotype 1	Frequency	Phenotypic variances^2^	SNPS
BLOCK1	111212	0.06	0.00	ALGA0003611|ASGA0002987|ALGA0003623|ALGA0003627|ALGA0003632|ALGA0003642
BLOCK1	112112	0.01	0.04	ALGA0003611|ASGA0002987|ALGA0003623|ALGA0003627|ALGA0003632|ALGA0003642
BLOCK1	111112	0.16	0.00	ALGA0003611|ASGA0002987|ALGA0003623|ALGA0003627|ALGA0003632|ALGA0003642
BLOCK1	112211	0.03	0.09	ALGA0003611|ASGA0002987|ALGA0003623|ALGA0003627|ALGA0003632|ALGA0003642
BLOCK1	111211	0.16	0.00	ALGA0003611|ASGA0002987|ALGA0003623|ALGA0003627|ALGA0003632|ALGA0003642
BLOCK1	112111	0.01	0.09	ALGA0003611|ASGA0002987|ALGA0003623|ALGA0003627|ALGA0003632|ALGA0003642
BLOCK1	111111	0.55	0.03	ALGA0003611|ASGA0002987|ALGA0003623|ALGA0003627|ALGA0003632|ALGA0003642
BLOCK2	2221222	0.22	0.29	ASGA0003043|MARC0003007|ASGA0003045|ASGA0003049|ASGA0003051|ALGA0003690|DRGA0000958
BLOCK2	2222122	0.19	0.48	ASGA0003043|MARC0003007|ASGA0003045|ASGA0003049|ASGA0003051|ALGA0003690|DRGA0000958
BLOCK2	2221122	0.14	0.00	ASGA0003043|MARC0003007|ASGA0003045|ASGA0003049|ASGA0003051|ALGA0003690|DRGA0000958
BLOCK2	2222222	0.35	0.00	ASGA0003043|MARC0003007|ASGA0003045|ASGA0003049|ASGA0003051|ALGA0003690|DRGA0000958
BLOCK2	2211222	0.02	0.00	ASGA0003043|MARC0003007|ASGA0003045|ASGA0003049|ASGA0003051|ALGA0003690|DRGA0000958
BLOCK2	2212222	0.03	0.04	ASGA0003043|MARC0003007|ASGA0003045|ASGA0003049|ASGA0003051|ALGA0003690|DRGA0000958
BLOCK2	2222221	0.01	0.19	ASGA0003043|MARC0003007|ASGA0003045|ASGA0003049|ASGA0003051|ALGA0003690|DRGA0000958
BLOCK2	2212221	0.01	0.01	H3GA0001822|ASGA0003063|ASGA0003062|ALGA0003699|ASGA0003070
BLOCK3	22111	0.03	0.07	H3GA0001822|ASGA0003063|ASGA0003062|ALGA0003699|ASGA0003070
BLOCK3	22211	0.15	0.27	H3GA0001822|ASGA0003063|ASGA0003062|ALGA0003699|ASGA0003070
BLOCK3	22121	0.05	0.03	H3GA0001822|ASGA0003063|ASGA0003062|ALGA0003699|ASGA0003070
BLOCK3	22221	0.16	0.02	H3GA0001822|ASGA0003063|ASGA0003062|ALGA0003699|ASGA0003070
BLOCK3	22112	0.04	0.26	H3GA0001822|ASGA0003063|ASGA0003062|ALGA0003699|ASGA0003070
BLOCK3	22212	0.12	0.00	H3GA0001822|ASGA0003063|ASGA0003062|ALGA0003699|ASGA0003070
BLOCK3	22122	0.10	0.12	H3GA0001822|ASGA0003063|ASGA0003062|ALGA0003699|ASGA0003070
BLOCK3	12222	0.02	0.04	H3GA0001822|ASGA0003063|ASGA0003062|ALGA0003699|ASGA0003070
BLOCK3	22222	0.32	0.01	H3GA0001822|ASGA0003063|ASGA0003062|ALGA0003699|ASGA0003070

1 : 1 is minor alleles and 2 is major allele.

2 : Percentage of deregressed EBV of total daily feed intake explained by markers based on association tests.

A total of 652 gene identities (Entrez ID) was located in 1Mb window size from SNP positions (Table S3). However, 283 genes were reported as repetitions, since they were located in overlapping regions between two or more windows. The final list of 369 genes with unique identity was used for functional annotation. The functional categories based on protein resource information (SP_PIR_KEYWORDS) and biological processes (GOTERM_BP_FAT) of nearby genes involved in feeding behavior are shown in Table 4.

**Table 4:** Functional annotation of nearby genes based on protein information and biological process.

Functional categories and gene ontologies	Terms	Gene names	P-value
SP_PIR_KEYWORDS	synapse	*GABRR2* 1, *PPP1R9B* 5, *SYT1* 3, *GABRR1* 1, *CADPS2* 4, *DLGAP2* 2, *GOPC* 1	0.01
SP_PIR_KEYWORDS	metalloprotein	*ACO1* 2, *ADH4* 5, *EPX, ADH5* 5, *MPO* 4	0.03
SP_PIR_KEYWORDS	protein phosphatase	*PPM1E* 4, *PTPN18* 6, *PTPRZ1* 4, *PTPN4* 4, *MTMR4* 4	0.03
GOTERM_BP_FAT	dephosphorylation	*PPM1E* 4, *DAPP1* 4, *PTPN18* 6, *PTPRZ1* 4, *PTPN4* 4, *MTMR4* 4, *RNGTT*	0.003
GOTERM_BP_FAT	positive regulation of peptide secretion	*GHRH* 6, *NNAT* 6, *TCF7L2* 4	0.02
GOTERM_BP_FAT	retinoid metabolic process	*SCPEP1* 4, *ADH4* 5, *ADH5* 5	0.02
GOTERM_BP_FAT	diterpenoid metabolic process	*SCPEP1* 4, *ADH4* 5, *ADH5* 5	0.02
GOTERM_BP_FAT	terpenoid metabolic process	*SCPEP1* 4, *ADH4* 5, *ADH5* 5	0.02

1 : Nearby genes to significant SNPs associated with total daily feed intake.

2 : Nearby genes to significant SNPs associated with mean feed intake per visit.

3 : Nearby genes to significant SNPs associated with mean feed intake rate.

4 : Nearby genes to significant SNPs associated with number of visits to the feeder per day.

5 : Nearby genes to significant SNPs associated with total time spent at feeder per day.

6 : Nearby genes to significant SNPs associated with time spent to eat per visit.

### Comparisons with previously mapped QTL in pigs

A total of 36 SNPs were located in the genomic region where QTLs have previously been mapped for behavior and/or feed intake traits in pigs (Table 5). Eight loci on SSC 1 and a locus on SSC 2 associated with DFI were located on previous QTL regions for feed intake/daily feed intake in other pig populations. Several significant SNPs for FR, FPV and TPD were found in QTL regions for drinking and socializing from previous studies. Moreover, we also detected five SNPs located in the genome regions where QTL/SNPs have been previously detected by GWAS for backfat traits in pigs.

**Table 5:** Comparative mapping of tag SNPs with previous QTLs reported in pig QTL database (Release 19, on Dec 27, 2012) and previous GWAS results.

Traits^1^	SNP	SSC^2^	SNP Position^3^ (bp)	Starting QTL Position^4^ (bp)	Ending QTL Position^5^ (bp)	QTL_ID^6^/reference	Corresponded Trait in QTL database
DFI	ASGA0003045	1	64018394	52874641	169149638	871	Feed intake
DFI	ASGA0003049	1	64036390	52874641	169149638	871	Feed intake
DFI	ASGA0003051	1	64054552	52874641	169149638	871	Feed intake
DFI	ALGA0003690	1	64094344	52874641	169149638	871	Feed intake
DFI	MARC0076100	1	64510071	52874641	169149638	871	Feed intake
DFI	ASGA0083328	1	64533206	52874641	169149638	871	Feed intake
DFI	H3GA0001822	1	64628578	52874641	169149638	871	Feed intake
FR	H3GA0006163	2	18828505	6419911	21506294	3889	Daily feed intake
FR	H3GA0006163	2	18828505	18710000	19500000	Fan et al, 2010 [71]	10th rib backfat
FR	MARC0098171	8	124871992	124156612	135387386	5947	Daily feed intake
FR	H3GA0025364	8	124894821	124156612	135387386	5947	Daily feed intake
TPD	ASGA0039757	8	128703259	124156612	135387386	5947	Daily feed intake
TPD	ALGA0049421	8	129335905	124156612	135387386	5947	Daily feed intake
TPD	H3GA0025421	8	129600171	124156612	135387386	5947	Daily feed intake
TPD	ASGA0039827	8	130796392	124156612	135387386	5947	Daily feed intake
TPD	ASGA0049581	11	6392619	3920148	31594979	5923	Time spent socializing
FR	ASGA00495811	11	6392619	3920148	31594979	5923	Time spent socializing
TPD	ALGA0060626	11	6443449	3920148	31594979	5923	Time spent socializing
TPD	M1GA0014839	11	6640240	3920148	31594979	5923	Time spent socializing
TPD	ALGA0060579	11	6845024	3920148	31594979	5923	Time spent socializing
TPD	ALGA0060596	11	7421327	3920148	31594979	5923	Time spent socializing
TPD	ASGA0049612	11	8505201	3920148	31594979	5923	Time spent socializing
TPD	ASGA0049606	11	8523653	3920148	31594979	5923	Time spent socializing
NVD	MARC0097496	12	39543788	38822400	47927603	5917	Time spent drinking
NVD	MARC0097496	12	39543788	38480000	38800000	Fan et al, 2010 [71]	10th rib backfat
TPV	ALGA0118892	12	60027710	61719816	61816078	3904	Average feeding rate
FPV	H3GA00383331	14	2744716	6898350	132053949	5722	Daily feed intake
NVD	MARC0080034	14	134634209	81745465	132170772	1164	Feed intake
NVD	ASGA0066557	14	134702823	81745465	132170772	1164	Feed intake
FPV	ALGA00826662	14	139614808	81745465	132170772	1164	Feed intake
FPV	ALGA00826662	14	139614808	139090000	139380000	Fan et al, 2010 [71]	last rib backfat
TPV	H3GA0054084	15	35839572	25021683	57165536	5915	Time spent drinking
FPV	MARC0104064	15	36548921	25021683	57165536	5915	Time spent drinking
FPV	ALGA0084813	15	37382667	25021683	57165536	5915	Time spent drinking
FPV	ALGA0090475	16	42490292	1167827	67649164	5953	Daily feed intake
FR	DRGA0017669	16	76276873	71797057	80266973	5918	Time spent drinking
NVD	ASGA0079300	18	26316841	26627380	–	Fontanesi et al, 2012 [30]	Backfat thickness
NVD	DRGA0016947	18	26825286	26627380	–	Fontanesi et al, 2012 [30]	Backfat thickness

1 : DFI: total daily feed intake, FPV: mean feed intake per visit, FR: mean feed intake rate, NVD: number of visits to the feeder per day, TPD: total time spent at feeder per day, TPV: time spent to eat per visit.

2 : Pig chromosome.

3 : SNP positions in Ensembl.

4 : Starting position of mapped QTL on QTL database.

5 : Ending position of mapped QTL on QTL database.

6 : Identity of QTL in pig QTL database or published literature.

### Comparative mapping of significant QTL with human genome

**Figure 4:**
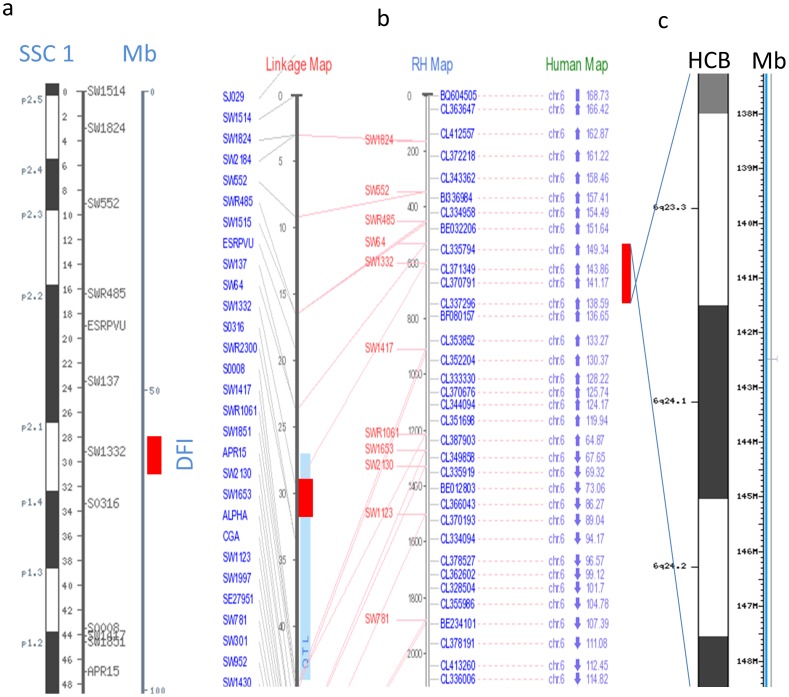
Comparative mapping between QTL on pig chromosome 1 and human chromosome 6. (**a**) Cytogenetic band, approximate positions of QTL shown in both cM and Mb, (b) linkage map, radiation hybrid mapping and human map of selected regions based on QTL database (release19), (c) human cytogentic band and physical map. The red band indicates QTL presence.

**Figure 5:**
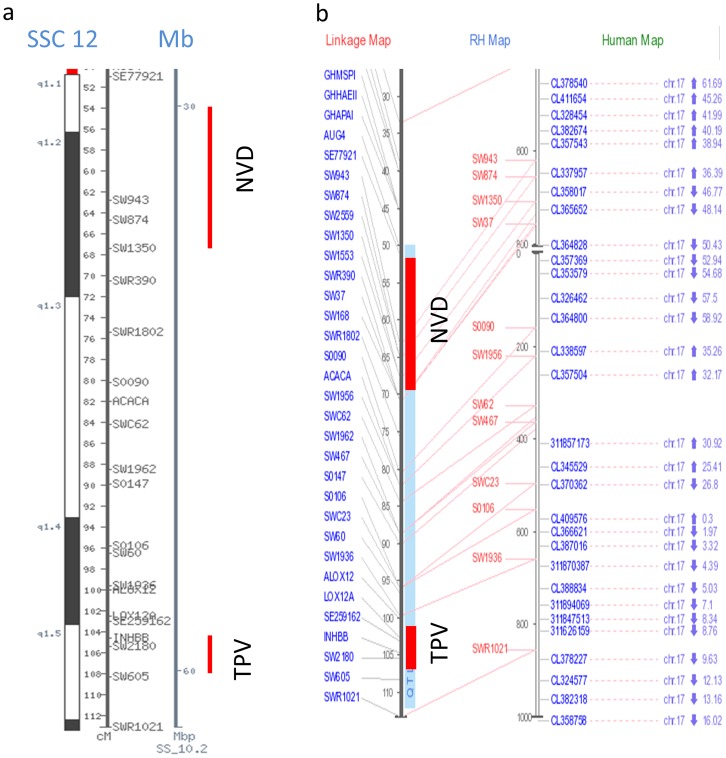
Comparative mapping between QTL on pig chromosome 12 and human chromosome 17. Cytogenetic band, approximate positions of QTL shown in both cM and Mb, (b) linkage map, radiation hybrid mapping and human map of selected regions based on QTL database (release19). The red band indicates QTL presence.

We indentified the five most significant QTL (contained more than 5 significant SNPs) for eating behavior traits including regions of 64–65 Mb on SSC 1 influencing DFI, 124–130 Mb on SSC 8 influencing both FR and TPD, 63–68 Mb on SSC 11 influencing TPD, 32–39 Mb and 59–60 Mb influencing NVD and TPV on SSC 12, respectively. The QTL region for DFI on SSC 1 located on p2.1 cytogenetic band (Figure 4a) which is homologous with the 136–157 Mb region on the human chromosome (HSA) 6 (Figure 4b and c). We also found that pleotropic QTL for FR and TPD on SSC 8 (124–130 Mb) was homologous with 90–101 Mb region on HSA 4 (HSA 4q22–24) (Figure S3). The QTL for TPD on SSC 11 was homologous with the 84–99Mb region on HSA13 (HSA 13q31–32) (Figure S4). Two QTL regions for NVD and TPV on SSC 12 located in q1.1–1.2 and q1.5 cytogenetic band (Figure 5a) were homologous with 36–48 Mbp (17q21 cytogentic band) and 4–8 Mb (17p13 cytogentic band) on HSA 17 (Figure 5b), respectively. Thus, our pig-human comparative mapping approaches revealed key genomic regions and/or genes on the human genome that may influence eating behavior in human beings and consequently obesity.

## Discussion

### Comparison with previously mapped QTL in pigs

Since no GWAS study for feeding behavior in pigs has been previously published, we have made an attempt to overlap our association signals with those of previously reported QTLs. However, direct comparison between data obtained in this study and those from previous QTL studies is hindered by the fact that locations given in centimorgan on different genome assemblies do not necessarily reflect the same physical location on the genome [26]. Therefore, the physical locations on the QTL (in Mb) as given in the SSC10.2 build in the pig QTLdb were used to compare to results from previous studies.

On SSC 1, we found that eight SNPs associated with DFI are in previously mapped QTL which spanned 49–73 (cM) for feed intake in a Pietrain/Meishan F2 family [27]. Moreover, we also found other SNPs associated with DFI very close to the QTL region mapped for DFI in full-sibs families based on cross-bred Pietrain, Large White, Landrace, and Leicoma [28]. This may imply that the same gene affected the traits across different pig breeds. On SSC 6, a QTL for TPV in pigs were also found on regions for time spent per day in a Pietrain x Meishan cross [27]. Other SNPs associated with feeding rate also found in QTL mapped for time spent feeding and socializing [27], drinking [27] and daily feed intake [29]. For instance, SNPs associated with FPV and TPD on SSC 8 were also found in the regions affecting DFI in Duroc×Petrain populations [29]. Because Lui *et al*. [29] did not find QTL for FPV and TPD, it is difficult to make any conclusions about pleiotropic effects of these QTL. Several SNPs associated with TPD on SSC 11 were also found in the QTL for time spent socializing in a Pietrain x Meishan cross [27]. Because the QTLs for fat deposition traits can be found over all pig chromosomes [30], we only compared our GWAS results with previous studies for backfat and obesity-related traits. Two SNPs associated with NVD on SSC18 in our study were found very close to a SNP detected for backfat thickness in an Italian breed [30]. Fontanesi *et al*, [30] found the neuronal genes play important roles in controlling fat deposition in this chromosome. These results suggested possible pleiotropic QTL/genes in the nervous system controlling both fat metabolism and feeding behavior. Some other QTLs and SNPs overlapping with previous studies might also be interesting for further investigation. Nevertheless, comparative mapping is useful for narrowing down QTL regions and targeting candidate genes for complex traits such as eating behavior.

### Haplotype block and haplotype frequency

Understanding linkage disequilibrium profiles and haplotype diversity in genomic regions of interest helps to better understand the genetic basis of these traits. The average LD observed in a Danish Duroc pig population was quite high (r^2^ = 0.56 between adjacent markers) [18]. High LD limits fine-mapping the QTL because of SNPs quite far from the actual QTL position, but it does not have much influence on an association test. In the candidate region (64–65 Mb) for DFI on SSC1, we found three haplotypes blocks with high LD between adjacent markers. An interesting haplotype block is 2222122 of seven markers including ASGA0003043, MARC0003007, ASGA0003045, ASGA0003049, ASGA0003051, ALGA0003690 and DRGA0000958 which contributed most (0.48 %) to phenotypic variance of DFI. Moreover, two SNPs in the haplotype were located in the intron region of two different genes (*GABRR2* and *SRSF12*); hence, it could be interesting to further investigate the functional involvement of these two genes in relation to DFI. Adjacent to GABRR2 is the *GABRR1* gene which encodes the GABA receptor γ1 subunit (Figure 2). In humans, *GABRR1* and *GABRR2* are highly linked and located in the GABA receptor cluster on SSC 6. Details of molecular functions and possible roles of *GABRR1* and *GABRR1* in relation to daily feed intake are discussed below. Furthermore, we also found that the haplotype 21222 for block 1 had the highest contribution to variances of NVD on SSC 12. All these SNPs were located in ankyrin-repeat and fibronectin type III domain containing the *ANKFN1* gene (Figure 3). *ANKFN1* was previously identified as a candidate gene in a genomic study of general vulnerability to substance use disorders in humans [31]. No functional investigations of the genes in pigs has been reported so far.

### Potential candidate genes

#### Potential candidate genes for average daily feed intake

Daily feed intake is an important trait for animal production and of general biological interest. Therefore, many studies have been conducted to investigate the genetic background underlying this trait. Only locus ALGA0003690 (G/A) was found to be significantly associated with DFI in the current study and it is located in the intron region of the Gamma-aminobutyric acid receptor subunit rho-2 (*GABRR2*) gene. *GABRR2* encodes for a receptor of Gamma-aminobutyric acid (GABA) which is the most important inhibitory neurotransmitter in the vertebrate central nervous system (CNS) and is involved in manifold physiological and pathological processes [32]. Moreover, a suggestive SNP associated with DFI was identified close to the *GABRR1* gene, which is in the same transcriptional orientation, suggesting a similar expression and regulatory pattern as *GABRR2*. The GABA and these receptors have a known function in controlling feed intake, as shown in different species such as rats [33], chickens [34], and ruminants [35]. Expression of *GABRR2* was significantly changed after fasting and refeeding in the hypothalamus in mice [36]. Baldwin *et al*. [37] showed that GABA and the GABA agonist stimulate feeding in satiated pigs by an action on central GABA receptors. However, the mechanism of GABA and these receptors in controlling feed intake and feed behavior is not well understood. Some other interesting genes in adjacent regions such as *SRSF12, ANKRD6, RRAGD, PM20D2, RNGGT, MDN1*, and *UBE2J1* might be interesting to investigate, since these functions are related to regulation of gene expression or signaling pathway (Table S3).

#### Potential candidate genes for time spent to eat per day

The significant loci MARC0085057 was closest to *ALX1* gene (Table 2), whose function has not been extensively studied even in humans. However, it is interesting to note that in a 1 Mb window around the SNP position we found the *NTS* gene which encodes a common precursor for two peptides, neuromedin N and neurotensin (Table S3). Neurotensin is a secreted tridecapeptide, which is widely distributed throughout the central nervous system and may function in controlling feeding behavior [38]. Intranigral microinjection of neurotensin suppressed feeding in food-deprived rats [39]. Nearby *DDIT4L* gene regulates the TOR signaling pathway and in turn mammalian target of rapamycin (mTOR) as a key fuel sensor in hypothalamic neurons [38]. Nutritional regulation of the mTOR-signaling pathway is mediated by their corresponding plasma membrane transporters [40]; therefore, *DDIT4L* may involve feeding behavior via nutritional impacts.

#### Potential candidate genes for number of visit to feeder per day

Two loci ASGA0054177 and M1GA0016584 had the strongest association with NVD (p = 9.65E-7 and 2.19E-6, respectively; Table 2). The musashi homolog 2 (*MIS2*) is the known gene located closest to them. This gene encodes an RNA-binding protein and play central roles in posttranscriptional gene regulation in mammals [41]. The gene also plays a role in the proliferation and maintenance of stem cells in the central nervous system in mice [42]. During neurogenesis, the MSI2 expression persisted in a subset of neuronal lineage cells, such as parvalbumin-containing GABA neurons in the neocortex [41]. As mentioned earlier, the GABA receptors also play a role in controlling feed intake and feeding behavior. It could be interesting to investigate how *MSI2* gene and *GABA* receptor genes are connected in controlling feeding behavior. Moreover, we found two mutations in the *TCF7L2* gene suggestively associated with NVD (Table S2). *TCF7L2* encodes for a transcriptional factor involved in Wnt signaling that can regulate the tumor necrosis factor-α induced antiadipogenesis, pancreatic β-cell survival and function [33] as well as primary immune response [43]. *TCF7L2* mutations were associated with backfat [35] and with meat color traits [44] and residual feed intake traits [45] in pigs.

#### Potential candidate gene for time spent to eat per visit

The ElaC homolog 2 (*ELAC2*) was close to significant SNPs associated with TPV (Table 2). This gene encodes for a protein which has a C-terminal domain with tRNA, processing endoribonuclease activity which catalyzes the removal of the 3′ trailer from precursor tRNAs. Mutations in this gene result in an increased risk of prostate cancer in humans [46]. No functional characterization of the gene in pigs are available so far. A mutation in intron regions of GATA binding protein 3 was suggested to be linked to TPV (Table S2). *GATA3* is a transcription factor of the Gata Zn-finger family which performs important functions during organogenesis [37]. In mice, the expression of gene was changed in obesity induced by different diet [47].

#### Potential candidate genes for feed intake per visit

The *ACO1* gene was close to significant SNPs associated with FPV (Table 2). The gene encodes for soluble aconitase, a bifunctional protein involved in the control of iron metabolism or as the cytoplasmic isoform of aconitase [48]. However, the gene has not been extensively studied in pigs. Disks large-associated protein 2 is a protein encoded by the *DLGAP2* gene (Table S2). The DLGAP2 protein is one of the membrane-associated guanylate kinases localized at postsynaptic density in neuronal cells [49] and may play a role in the molecular organization of synapses and in neuronal cell signaling. The *DLGAP2* variants were found significantly associated with autism spectrum disorders [50]. Ceroid-lipofuscinosis neuronal 8 (*CLN8*) plays a role in cell proliferation during neuronal differentiation [51]. Both *DLGAP2* and *CLN8* were located on SSC15 (Table S2) and may be of interest for feeding behavior traits, because it functions in the neuronal center controlling feed intake.

#### Potential candidate genes for rate of feed intake

The *PPA2* gene may be an interesting candidate gene for rate of feed intake, since two variants of the gene were found suggestively associated with the trait. The protein encoded by this gene is localized to the mitochondrion and contains the signature sequence essential for the catalytic activity of PPase [52]. *PPA2* may have a function in feeding behavior via controlling the phosphate level of the cell. Neuromedin U Receptor 2 (*NMU2*) is the most interesting gene for FR (Table S2). Neuromedin U is a known neuropeptide with potent activity on smooth muscle which is widely distributed in the gut and central nervous system [53]. The *NMUR2* gene is expressed in the ventromedial hypothalamus in the rat brain and its level is significantly reduced following fasting [54]. Neuromedin U receptor 2-deficient mice display differential responses in sensory perception, stress, and feeding [55].

### Functional categories of potential candidate genes

The results of functional annotation of nearby genes showed many genes involved in synapses that are essential to neuronal functions. The *GABRR2, PPP1R9B, SYT1, GABRR1, CADPS2, DLGAP2 and GOPC* genes were involved in activities for synapses based on protein resource information (Table 4). Functions of *DLGAP2, GABRR1 and GABRR2* in feeding behavior have been discussed above. In humans, *SYT1* encodes for Synaptotagmin-1 protein SYT1 which is the master switch responsible for allowing the human brain to release neurotransmitters [56]. Protein encoded by protein phosphatase 1, regulatory subunit 9B (*PPP1R9B*) plays an important role in linking the actin cytoskeleton to the plasma membrane at the synaptic junction [57]. The *CADPS2* gene encodes a member of the calcium-dependent activator of secretion (CAPS) protein family, which are calcium-binding proteins that regulate the exocytosis of synaptic and dense-core vesicles in neurons [58]. Dephosphorylation is the essential process of removing phosphate groups from an organic compound as adenosine triphosphates (ATP) by hydrolysis. Feeding behavior has been linked to ATP concentration in the liver with satiety occurring as fuels are oxidized and ATP is produced, and hunger occurring as oxidation decreases and ATP is depleted [59]. Seven nearby genes have been classified in dephosphorylation based on their functions and may play significant roles in this mechanism (Table 4). The *PTPN4, PTPN18* and *PTPRZ1* genes are members of the protein tyrosine phosphatase (PTP) family. A recent review described PTPs as central regulators of metabolism, specifically highlighting their interactions with the neuronal leptin and insulin signaling pathways [60]. On the other hand, *PPM1E* was located in the nucleus of the cell and it encodes a member of the PPM family of serine/threonine-protein phosphatases. The encoded protein dephosphorylates and inactivates multiple substrates such as 5′-AMP-activated protein kinase (AMPK) which is well documented to play key roles in controlling energy balance [61]. AMPK appears to play a role in hypothalamic glucose and nutrient sensing [61]. Therefore, the function of the *PPM1E* gene on feeding behavior may be mediated by AMPK. Another significant biological process involves the nearby genes (*GHRH, NNAT, and TCF7L2*) having a positive regulation of peptide secretion (Table 4). Growth hormone-releasing hormone (GHRH) is well known to stimulate food intake [62] and will therefore not be discussed further. The *TCF7L2* has been proven as candidate gene for residual feed intake, as discussed above.

### Implications for humans by comparative QTL/genomic mapping

Our pig-human comparative mapping approaches revealed key genomic regions and/or genes on the human genome that may influence eating behavior in human beings and consequently lead to obesity and metabolic syndrome. For instance, the QTL for DFI on SSC 1 was homologous with HSA 6q23–24 region (Figure 4a and b) which has been found to significantly affect obesity-related traits in humans such as waist circumference, body mass index or fasting glucose and insulin levels in different studies (reviewed in [63]). The region also contains several genes associted with obesity or metabolic syndrome such as *ENPP1* with obesity and risk of glucose intolerance and type 2 diabetes [64], *SGK1* with insulin secretion in type 2 diabeties [65]. Frequency of eating and meal time are important indicators for eating behavior in humans. QTL for NVD was homologous with HSA 17q21 regions which contained many obesity candidate genes including *PPY*, *PON1* and 2, *GAST, PNMT, STAT3* and *HCRT* (reviewed in [63]). Moreover, some of the genes have been found to play very important roles in controlling feed intake in both human and animal models. For instance, the *HCRT* gene encodes a hypothalamic neuropeptide precursor protein that gives rise to two mature neuropeptides, orexin A and orexin B, which stimulate feed intake in rats [66]. Peptide YY (*PYY*) also plays a very important role in energy homeostasis by balancing food intake [67] by acting as an “ileal brake” leading to a sensation of fullness and satiety [68]. Other homologous regions including HSA 4q22–24, HSA 13q31–32 and HSA 17p13 also contain a number of candidate genes for obesity/ metabolic syndorome and eating behavior in both human and animals. For instance, microsomal triglyceride transfer protein (*MMTP*) gene located in HSA q24 were found as a candidate gene for obesity [63] in humans. The inhibitation of this gene by JTT-30 was found to suppress also the food intake in rats [69]. The function of the *MTTP* gene in feed intake may be due to its involvement in the gut leptin-melanocortin pathway [70]. Although pigs and humans have similar genetic structure, comparative genomic mapping between these species has a limitation on accuracy of homolgous regions. This limitation can be overcome by fine mapping or meta-analysis of QTL in each species and by taking systems biology approaches that links genomic regions with phenotypes through transcriptomics to detect potential causal genes ([5]–[6] and [43]). Nevertheless, the results of comparative QTL mapping from this study are useful for understanding the genetic background of eating behavior in humans (more QTL for traits) as well as in pigs (more candidate genes with functional validations).

## Conclusion

Feeding or eating behavior are important traits in pig production, as they are directly related to feed efficiency and hence cost of pig production, but their genetic mechanisms have not been extensively studied. This is the first GWAS study pinpointing a number of significant SNPs associated with feeding or eating behavior in pigs. This study presented a comprehensive approach by combining GWAS and post-GWAS bioinformatics as well as comparative mapping approaches to elucidate genomic regions and candidate genes associated with eating behavioral traits in pigs. Post-GWAS analyses highlighted potential candidate genes for feeding behavior. Several nearby genes have been mentioned directly or indirectly as being involved in the genetic control of eating or feeding behavior traits in either pigs or other species. Pigs are a well-known animal model for studying human obesity. We have conducted pig-human comparative gene mapping to reveal key genomic regions and/or genes on the human genome that may influence eating behavior in human beings and consequently affect the development of obesity and metabolic syndrome, both of which are key societal and public health problems. This is the first study to report results on genes that may affect human eating behavior via such translational genomics approaches.

## Supporting Information

Figure S1 **Multidimensional scaling plot of identity by state distances.** The principal component analysis fitted the genetic distances along the two components. The results showed that no population stratification in the data. Each point on the plot corresponds to a pig, and the 2D distances between points were fitted to be as close as possible to those presented in the original identity by state matrix. You can see that study subjects clearly cluster in a group.
(TIF)

Figure S2 **A quantile-quantile plot of observed and expected** ***p-*** **values for feeding behavior traits.** The inset shows a quantile-quantile (qq) plot with the observed plotted against the expected p-values for total daily feed intake (DFI), total time spent at feeder per day (TPD), number of visits to the feeder per day (NVD), time spent to eat per visit (TPV), mean feed intake per visit (FPV), and mean feed intake rate (FR) from top to bottom, respectively.
(TIF)

Figure S3 **Comparative mapping between QTL on pig chromosome 8 and human** **chromosome 4.** (**a**) Cytogenetic band, approximate positions of QTL for mean of feed intake rate FR) and total time spent at feeder per day (TPD) shown in both cM and Mb, (b) linkage map, radiation hybrid mapping and human map of selected regions based on QTL database (release19). The read band indicated QTL presence.
(TIF)

Figure S4 **Comparative mapping between QTL on pig chromosome 11 and human** **chromosome 13.** (**a**) Cytogenetic band, approximate positions of QTL for total time spent at feeder per day (TPD) shown in both cM and Mb, (b) linkage map, radiation hybrid mapping and human map of selected regions based on QTL database (release19). The red band indicates QTL presence.
(TIF)

Table S1 **Distribution of SNPs after quality control and average distances on each chromosome.**
(DOC)

Table S2 **Suggestive SNPs associated to studied eating behavioral traits, their positions and nearest genes for feeding behavior traits.**
(DOC)

Table S3 **List of nearby genes in 1 Mb region flanking the associated SNPs.**
(DOC)

Table S4 **Haplotypes and their frequencies in the candidate region for number of visits to feeder per day on chromosome 12.**
(DOC)
