# Supplementary figures and images for: Genome-Wide Association Study Reveals Genetic Architecture of Eating Behavior in Pigs and Its Implications for Humans Obesity by Comparative Mapping
Source: PLoS One. 2013 Aug 19;8(8):e71509. doi: 10.1371/journal.pone.0071509 (PMC3747221; doi:10.1371/journal.pone.0071509)

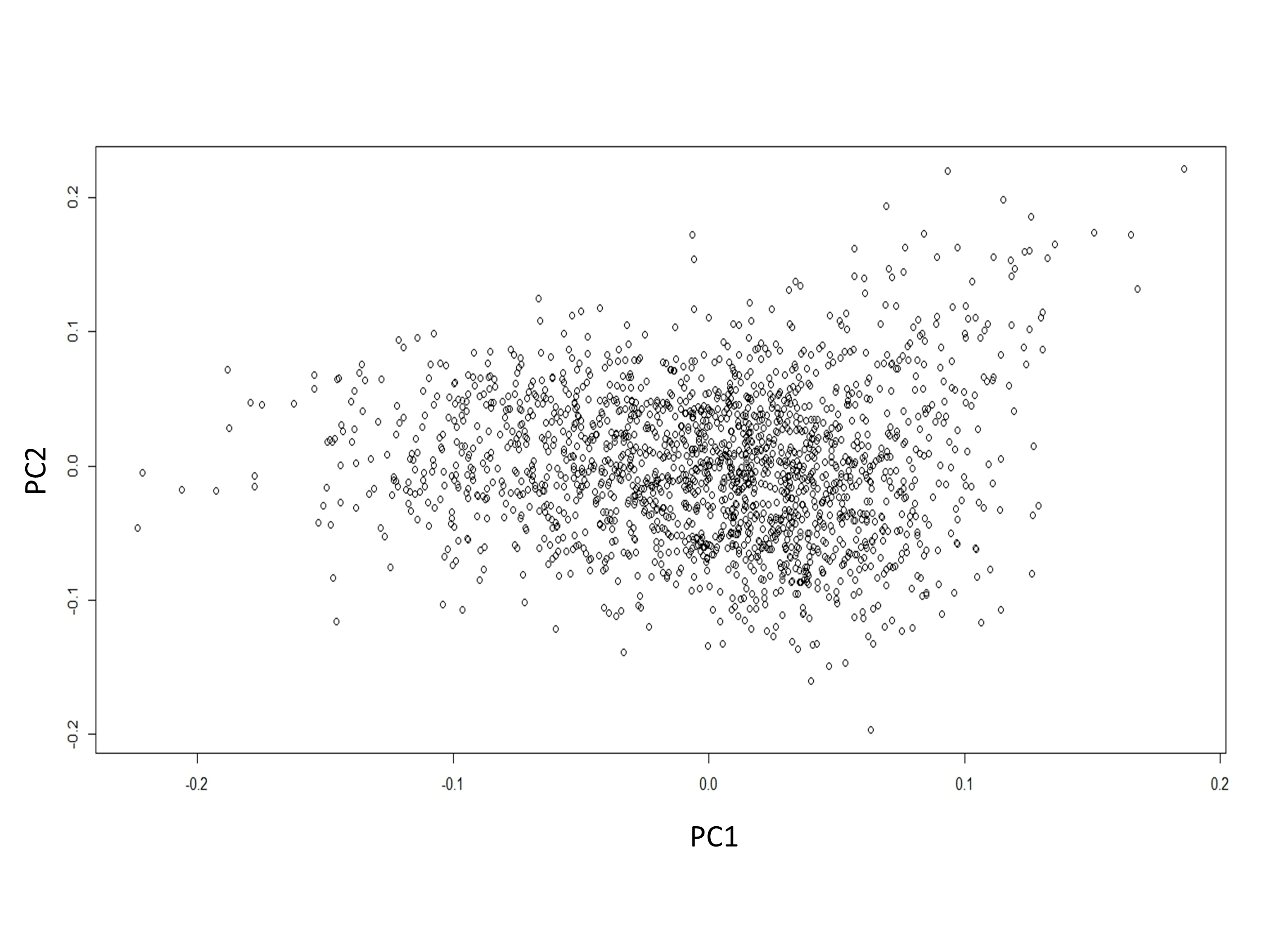

Supplement: Figure S1 — Multidimensional scaling plot of identity by state distances. The principal component analysis fitted the genetic distances along the two components. The results showed that no population stratification in the data. Each point on the plot corresponds to a pig, and the 2D distances between points were fitted to be as close as possible to those presented in the original identity by state matrix. You can see that study subjects clearly cluster in a group. (TIF) [file pone.0071509.s001.tif]

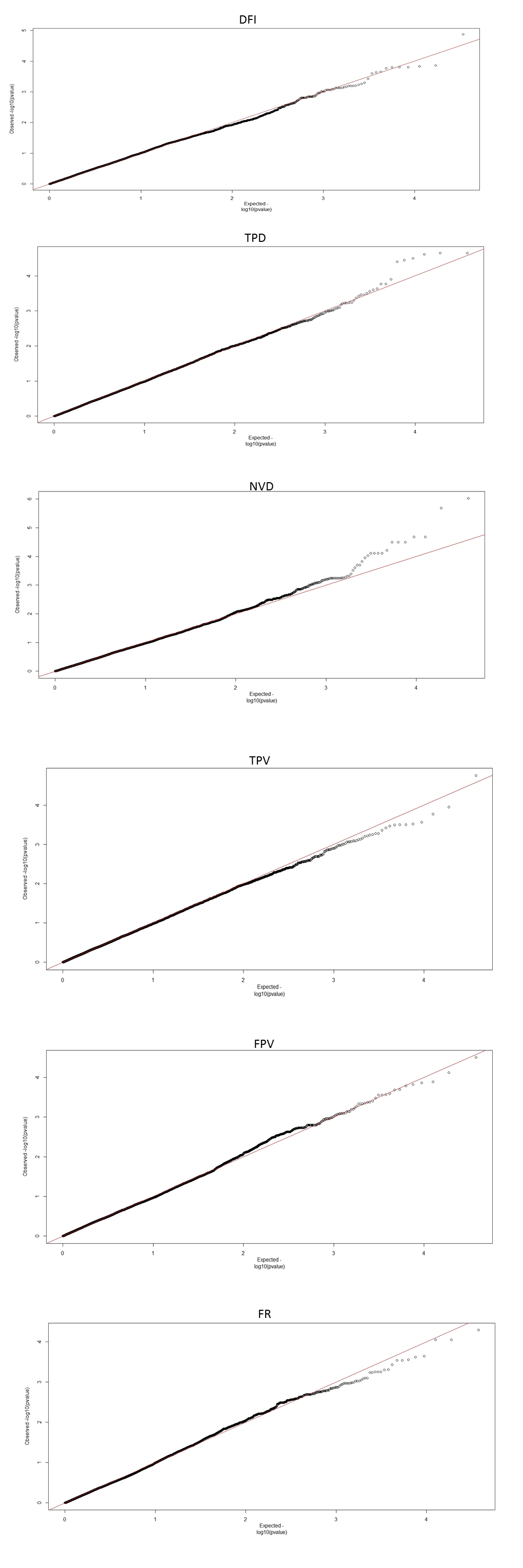

Supplement: Figure S2 — A quantile-quantile plot of observed and expected p- values for feeding behavior traits. The inset shows a quantile-quantile (qq) plot with the observed plotted against the expected p-values for total daily feed intake (DFI), total time spent at feeder per day (TPD), number of visits to the feeder per day (NVD), time spent to eat per visit (TPV), mean feed intake per visit (FPV), and mean feed intake rate (FR) from top to bottom, respectively. (TIF) [file pone.0071509.s002.tif]

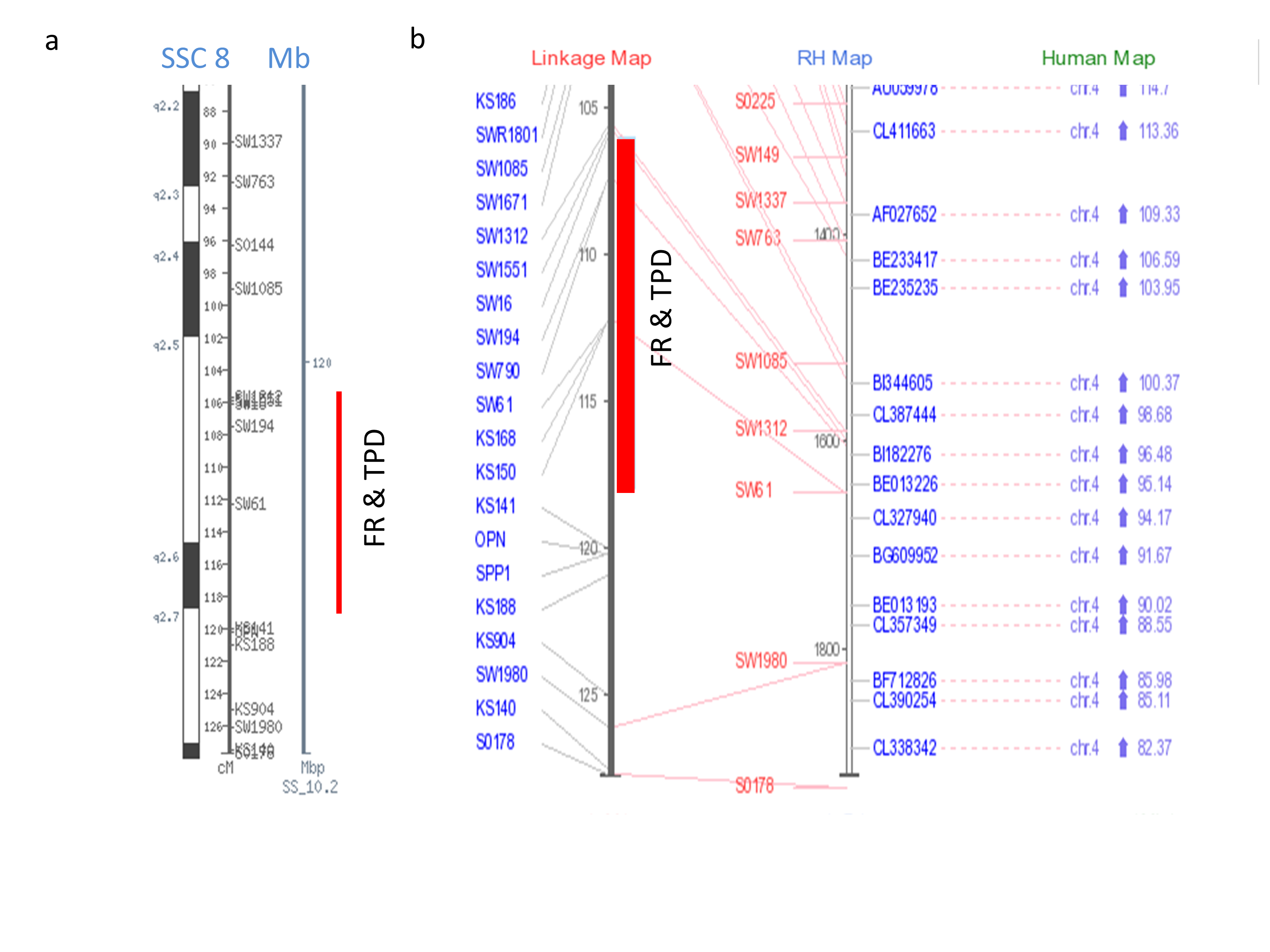

Supplement: Figure S3 — Comparative mapping between QTL on pig chromosome 8 and human chromosome 4. (a) Cytogenetic band, approximate positions of QTL for mean of feed intake rate FR) and total time spent at feeder per day (TPD) shown in both cM and Mb, (b) linkage map, radiation hybrid mapping and human map of selected regions based on QTL database (release19). The read band indicated QTL presence. (TIF) [file pone.0071509.s003.tif]

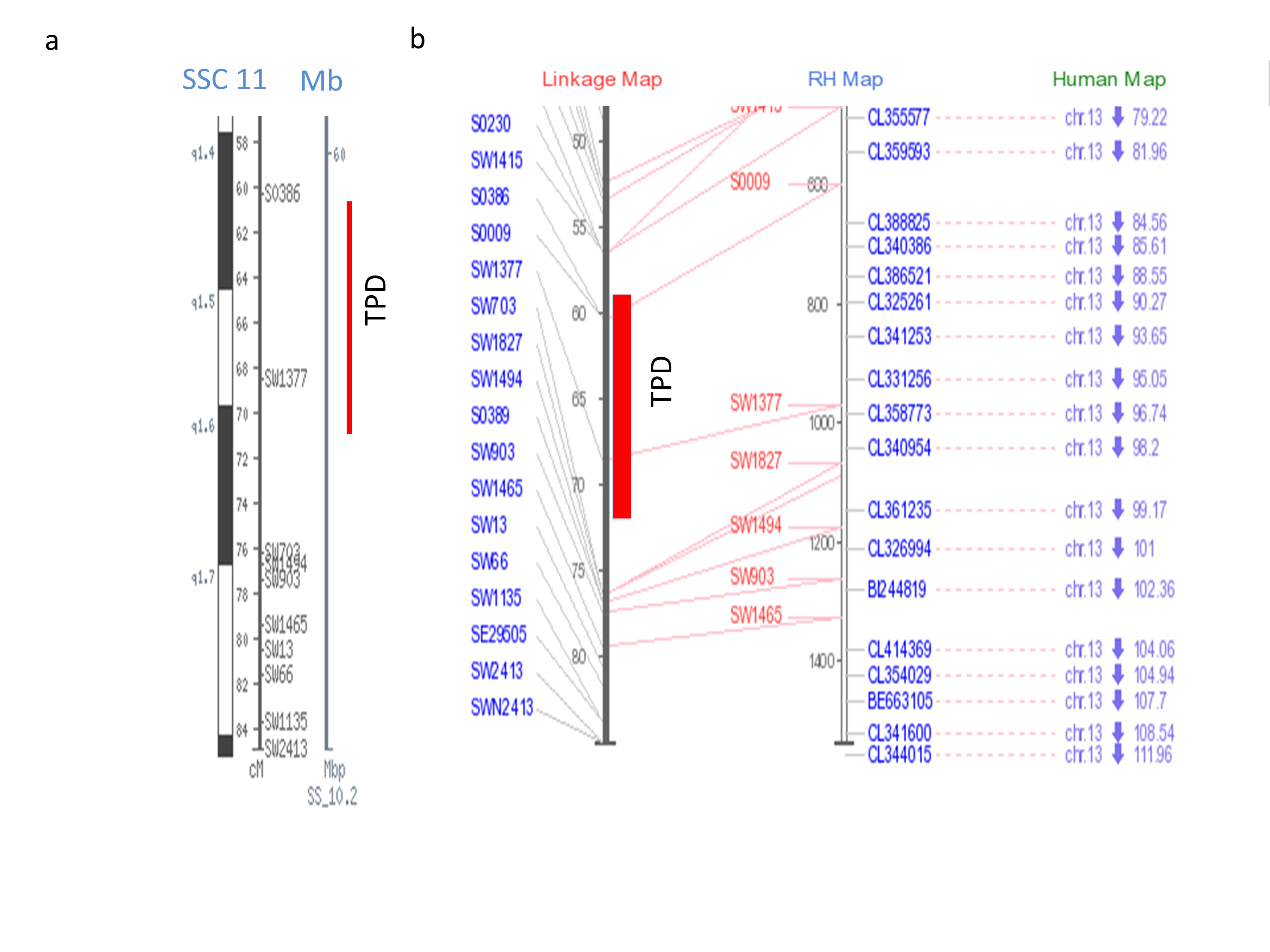

Supplement: Figure S4 — Comparative mapping between QTL on pig chromosome 11 and human chromosome 13. (a) Cytogenetic band, approximate positions of QTL for total time spent at feeder per day (TPD) shown in both cM and Mb, (b) linkage map, radiation hybrid mapping and human map of selected regions based on QTL database (release19). The red band indicates QTL presence. (TIF) [file pone.0071509.s004.tif]
