# Supplementary material for: Genome-Wide Association Study Reveals Genetic Architecture of Eating Behavior in Pigs and Its Implications for Humans Obesity by Comparative Mapping
Source: PLoS One. 2013 Aug 19;8(8):e71509. doi: 10.1371/journal.pone.0071509 (PMC3747221; doi:10.1371/journal.pone.0071509)
Supplement: Table S1 — Distribution of SNPs after quality control and average distances on each chromosome. (DOC) [file pone.0071509.s005.doc]

**Table S1. Distribution of SNPs after quality control and average distances on each chromosome**

| SSC | No.SNPs | Average marker distance (kb)^1^ |
| --- | --- | --- |
| 1 | 2904 | 108.58 |
| 2 | 1612 | 100.85 |
| 3 | 1137 | 127.34 |
| 4 | 2255 | 63.62 |
| 5 | 1152 | 96.79 |
| 6 | 1152 | 136.95 |
| 7 | 1986 | 67.86 |
| 8 | 1316 | 112.84 |
| 9 | 1381 | 111.27 |
| 10 | 809 | 97.78 |
| 11 | 1117 | 78.51 |
| 12 | 654 | 97.23 |
| 13 | 2022 | 108.13 |
| 14 | 2335 | 65.89 |
| 15 | 1426 | 110.58 |
| 16 | 952 | 91.28 |
| 17 | 902 | 77.27 |
| 18 | 657 | 93.18 |
| X | 51 | 2829.18 |
| 0^2^ | 8125 |  |
| Total | 33945 |  |

^1^Derived from Sus scrofa Build 10.2

^2^These SNPs are not assigned to a specific chromosome according to Illumina PorcineSNP60 Beadchip.
