# Supplementary material for: Genome-Wide Association Study Reveals Genetic Architecture of Eating Behavior in Pigs and Its Implications for Humans Obesity by Comparative Mapping
Source: PLoS One. 2013 Aug 19;8(8):e71509. doi: 10.1371/journal.pone.0071509 (PMC3747221; doi:10.1371/journal.pone.0071509)
Supplement: Table S2 — Suggestive SNPs associated to studied eating behavioral traits, their positions and nearest genes for feeding behavior traits. (DOC) [file pone.0071509.s006.doc]

**Table S2**. Suggestive SNP associated to studied traits, their positions and nearest genes and distance from SNPs to corresponded genes

| **Trait^1^** | **SNP** | **SSC^2^** | **Position** | **Ensembl Gene ID** | **Gene** | **Distances (bp)^3^** | **P_GC_^4^** | **P_raw_^5^** |
| --- | --- | --- | --- | --- | --- | --- | --- | --- |
| DFI | H3GA0001822 | 1 | 64628578 | *ENSSSCG00000004328* |  | intron | 1.38E-04 | 5.68E-06 |
| DFI | MARC0076100 | 1 | 64510071 | *ENSSSCG00000004322* | *ANKRD6* | intron | 1.59E-04 | 3.51E-07 |
| DFI | ASGA0083328 | 1 | 64533206 | *ENSSSCG00000004322* | *ANKRD6* | intron | 1.59E-04 | 3.51E-07 |
| DFI | H3GA0001815 | 1 | 49380268 | *ENSSSCG00000020885* | *NUS1* | 121406 | 1.59E-04 | 3.51E-07 |
| DFI | ASGA0003051 | 1 | 64054552 | *ENSSSCG00000024249* | *GABRR2* | 10755 | 1.73E-04 | 1.09E-05 |
| DFI | ASGA0003045 | 1 | 64018394 | *ENSSSCG00000004315* | *GABRR1* | -17550 | 2.30E-04 | 1.46E-05 |
| DFI | ASGA0003049 | 1 | 64036390 | *ENSSSCG00000004315* | *GABRR1* | -35546 | 2.49E-04 | 1.56E-05 |
| DFI | CAIL0000146 | 2 | 142136584 | *ENSSSCG00000014298* | *PPP2CB* | -20843 | 1.47E-04 | 4.41E-05 |
| DFI | ALGA0111383 | 9 | 118454584 | *ENSSSCG00000015444* | *LAMB1* | intron | 2.23E-04 | 9.94E-06 |
| DFI | ALGA0058575 | 10 | 44428078 | *ENSSSCG00000011014* | *BAMBI* | -186474 | 3.79E-04 | 3.71E-05 |
| NVD | MARC0030253 | NA | NA | *NA* |  |  | 7.95E-05 | 8.37E-08 |
| NVD | H3GA0029068 | 10 | 5156226 | *ENSSSCG00000010809* | *FAM5C* | -162697 | 4.19E-04 | 4.19E-03 |
| NVD | ASGA0084073 | 12 | 34087275 | *ENSSSCG00000017614* | *TRIM25* | intron | 7.95E-05 | 8.37E-08 |
| NVD | MARC0045984 | 12 | 34151283 | *ENSSSCG00000017614* | *TRIM25* | -70354 | 7.95E-05 | 8.37E-08 |
| NVD | ASGA0054137 | 12 | 33907320 | *ENSSSCG00000030781* | *C17orf67* | 23732 | 7.95E-05 | 8.37E-08 |
| NVD | ASGA0054337 | 12 | 36387623 | *ENSSSCG00000029046* |  | 1748 | 1.01E-04 | 1.65E-07 |
| NVD | ALGA0065784 | 12 | 26506162 | *ENSSSCG00000017580* | *TMEM92* | -8766 | 1.52E-04 | 3.67E-06 |
| NVD | H3GA0034044 | 12 | 32074865 | *ENSSSCG00000030875* | *STXBP4* | 41812 | 2.41E-04 | 9.73E-07 |
| NVD | MARC0011225 | 12 | 32136826 | *ENSSSCG00000030875* | *STXBP4* | -20149 | 2.41E-04 | 9.73E-07 |
| NVD | ASGA0066557 | 14 | 134702823 | *ENSSSCG00000010638* | *TCF7L2* | intron | 1.80E-04 | 1.80E-04 |
| NVD | H3GA0038333 | 14 | 2865914 | *ENSSSCG00000009589* | *SYK* | 338 | 2.87E-04 | 2.87E-05 |
| NVD | MARC0080034 | 14 | 134634209 | *ENSSSCG00000010638* | *TCF7L2* | intron | 5.00E-04 | 5.00E-05 |
| NVD | ALGA0092509 | 17 | 509344 | *ENSSSCG00000020727* |  | -32169 | 8.01E-05 | 8.01E-04 |
| NVD | ASGA0079300 | 18 | 26316841 | *ENSSSCG00000016611* | *CADPS2* | intron | 3.84E-04 | 4.60E-05 |
| NVD | DRGA0016947 | 18 | 26825286 | *ENSSSCG00000016614* | *PTPRZ1* | -174089 | 4.82E-04 | 2.26E-05 |
| TPD | ASGA0105377 | NA | NA | NA |  |  | 2.51E-04 | 6.51E-06 |
| TPD | M1GA0026294 | NA | NA | NA |  |  | 3.50E-04 | 4.23E-04 |
| TPD | ASGA0095686 | NA | NA | NA |  |  | 3.77E-04 | 1.70E-04 |
| TPD | INRA0020528 | 5 | 102326523 | *ENSSSCG00000000936* | *SLC6A15* | -216634 | 2.34E-04 | 3.50E-04 |
| TPD | ALGA0049421 | 8 | 129335905 | *ENSSSCG00000025240* | *DDIT4L* | 214492 | 1.69E-04 | 2.91E-05 |
| TPD | H3GA0025421 | 8 | 129600171 | *ENSSSCG00000030133* |  | 15270 | 1.69E-04 | 2.91E-05 |
| TPD | ASGA0039757 | 8 | 128703259 | *ENSSSCG0000000917* | *PPP3CA* | intron | 2.77E-04 | 4.23E-04 |
| TPD | ASGA0039827 | 8 | 130796392 | *ENSSSCG00000029813* | *TSPAN5* | 46995 | 3.15E-04 | 2.89E-05 |
| TPD | M1GA0014839 | 11 | 6640240 | *ENSSSCG00000009326* | *KATNAL1* | 120377 | 1.26E-04 | 1.96E-08 |
| TPD | ALGA0060579 | 11 | 6845024 | *ENSSSCG00000009326* | *KATNAL1* | -84407 | 3.49E-04 | 3.83E-08 |
| TPD | ASGA0068413 | 15 | 7579316 | *ENSSSCG00000021343* | *ZEB2* | 366985 | 4.23E-04 | 5.14E-04 |
| TPV | MARC0053050 | 6 | 29469612 | *ENSSSCG00000002835* | *TOX3* | -163014 | 1.70E-04 | 3.17E-04 |
| TPV | ALGA0103136 | 6 | 30028044 | *ENSSSCG00000002836* | *SALL1* | 194437 | 2.73E-04 | 5.93E-07 |
| TPV | ALGA0035106 | 6 | 30009402 | *ENSSSCG00000002836* | *SALL1* | 213079 | 3.80E-04 | 1.45E-06 |
| TPV | MARC0091414 | 10 | 69589046 | *ENSSSCG00000011125* | *GATA3* | intron | 1.11E-04 | 5.79E-07 |
| TPV | ALGA0118892 | 12 | 60027710 | *ENSSSCG00000028465* | *ELAC2* | 69073 | 3.14E-04 | 7.49E-04 |
| TPV | ALGA0102886 | 12 | 59982065 | *ENSSSCG00000028465* | *ELAC2* | 114718 | 3.17E-04 | 7.23E-04 |
| TPV | ASGA0100785 | 12 | 59966291 | *ENSSSCG00000028465* | *ELAC2* | 130492 | 3.41E-04 | 6.70E-04 |
| TPV | H3GA0054084 | 15 | 35839572 | *ENSSSCG00000015734* | *IMP4* | -43645 | 3.05E-04 | 4.11E-05 |
| TPV | ALGA0095059 | 17 | 45969331 | *ENSSSCG00000030835* | *BLCAP* | 70400 | 4.40E-04 | 3.38E-06 |
| TPV | DRGA00169471 | 18 | 13583864 | *ENSSSCG00000027601* | *FAM180A* | 723849 | 3.21E-04 | 6.70E-05 |
| FPV | ALGA01028861 | NA | NA | NA |  |  | 1.38E-04 | 3.84E-04 |
| FPV | ALGA01188921 | NA | NA | NA |  |  | 1.50E-04 | 4.82E-04 |
| FPV | ASGA01007851 | NA | NA | NA |  |  | 1.64E-04 | 2.51E-04 |
| FPV | M1GA00245242 | NA | NA | NA |  |  | 3.29E-04 | 3.50E-04 |
| FPV | DRGA0017416 | NA | NA | NA |  |  | 4.20E-04 | 2.77E-04 |
| FPV | ALGA0102707 | NA | NA | NA |  |  | 4.65E-04 | 3.81E-05 |
| FPV | ALGA0042544 | 7 | 78311813 | ENSSSCG00000024396 | *NOVA1* | *-309314* | 4.65E-04 | 3.81E-05 |
| FPV | MARC0001935 | 7 | 77275077 | *ENSSSCG00000024396* | *NOVA1* | 727422 | 4.65E-04 | 3.81E-05 |
| FPV | M1GA00143421 | 10 | 38849933 | *ENSSSCG00000023807* | *ACO1* | -655081 | 7.58E-05 | 6.17E-08 |
| FPV | ASGA0048906 | 10 | 70074394 | *ENSSSCG00000021720* |  | intron | 2.73E-04 | 2.73E-04 |
| FPV | H3GA00383331 | 14 | 2744716 | *ENSSSCG00000028560* | *DIRAS2* | 93904 | 1.31E-04 | 3.80E-04 |
| FPV | ALGA00826662 | 14 | 139614808 | *ENSSSCG00000010672* | *RAB11FIP2* | intron | 4.00E-04 | 1.11E-04 |
| FPV | ALGA0084813 | 15 | 37382667 | *ENSSSCG00000015746* | *DLGAP2* | 272106 | 4.30E-04 | 3.14E-04 |
| FPV | MARC0104064 | 15 | 36548921 | *ENSSSCG00000022715* |  | 25605 | 4.49E-04 | 3.17E-04 |
| FPV | ALGA0090475 | 16 | 42490292 | *ENSSSCG00000016933* | *ELOVL7* | 12070 | 2.08E-04 | 6.41E-06 |
| FPV | ASGA00793002 | 18 | 13082762 | *ENSSSCG00000016523* |  | -29008 | 2.03E-04 | 2.39E-04 |
| FPV | DRGA00169472 | 18 | 13583864 | *ENSSSCG00000016523* |  | -530110 | 2.58E-04 | 2.92E-04 |
| FPV | ALGA00975731 | 18 | 13446026 | *ENSSSCG00000016523* |  | -392272 | 2.80E-04 | 2.92E-04 |
| FPV | ASGA00793331 | 18 | 13503866 | *ENSSSCG00000016523* |  | -450112 | 2.80E-04 | 4.94E-04 |
| FR | ASGA0089165 | NA | NA | *NA* |  |  | 9.00E-05 | 1.92E-06 |
| FR | ASGA0007170 | 1 | 289023540 | *ENSSSCG00000027360* |  | 135949 | 2.26E-04 | 1.94E-05 |
| FR | ASGA0007175 | 1 | 289350190 | *ENSSSCG00000027360* |  | intron | 2.77E-04 | 2.45E-05 |
| FR | DRGA0006450 | 5 | 107536603 | *ENSSSCG00000023494* |  | 493490 | 2.39E-04 | 9.36E-06 |
| FR | H3GA0025364 | 8 | 124894821 | *ENSSSCG00000022365* | *PPA2* | intron | 2.92E-04 | 1.39E-05 |
| FR | MARC0098171 | 8 | 124871992 | *ENSSSCG00000022365* | *PPA2* | intron | 2.92E-04 | 1.39E-05 |
| FR | DRGA00087791 | 8 | 57141466 | *ENSSSCG00000029636* |  | 38451 | 4.94E-04 | 4.33E-06 |
| FR | ALGA00493341 | 8 | 57187784 | *ENSSSCG00000029636* |  | -7867 | 4.97E-04 | 4.13E-06 |
| FR | ASGA00495811 | 11 | 6392619 | *ENSSSCG00000009324* | *SLC7A1* | -75095 | 3.71E-04 | 3.78E-08 |
| FR | DRGA0017669 | 16 | 76276873 | *ENSSSCG00000017076* | *NMUR2* | 246207 | 9.00E-05 | 1.92E-06 |

^1^ DFI: total daily feed intake, FPV: mean feed intake per visit, FR: mean feed intake rate, NVD: number of visits to the feeder per day, TPD: total time spent at feeder per day, TPV: time spent to eat per visit

^2^ SNP names according to Illumina- Porcine beadchip

^3^ Pig chromosomes, NA = Position is not available in SSC10.2

^4^ Distance from SNPs to starting point of genes

^5^P_GC_ : GWAS p-value after genomic control

^6^ P_raw_: GWAS p-value before genomic control
