# Supplementary material for: Genome-Wide Association Study Reveals Genetic Architecture of Eating Behavior in Pigs and Its Implications for Humans Obesity by Comparative Mapping
Source: PLoS One. 2013 Aug 19;8(8):e71509. doi: 10.1371/journal.pone.0071509 (PMC3747221; doi:10.1371/journal.pone.0071509)
Supplement: Table S3 — List of nearby genes in 1 Mb region flanking the associated SNPs. (DOC) [file pone.0071509.s007.doc]

**Table S3: The candidate genes within a 1Mb window from SNP position**

| **Trait^1^** | **SNP** | **SSC** | **Position** | **1Mb-bin- gene^2^** |
| --- | --- | --- | --- | --- |
| DFI | ALGA0003690 | 1 | 64094344 | *SRSF12, LOC100153533, GABRR1, LOC100156383, RRAGD, PM20D2, LOC100520705, RNGTT, LOC100522289, LOC100523036, LOC100524470, MDN1, LOC100627571, LOC100739281, LOC100739399, LOC100739554, LOC100739627, UBE2J1* |
| DFI | ASGA0003045 | 1 | 64018394 | *SRSF12, LOC100153533, GABRR1, LOC100156383, RRAGD, PM20D2, LOC100520705, RNGTT, LOC100522289, LOC100524470, LOC100739281, LOC100739399, LOC100739554, LOC100739627, UBE2J1* |
| DFI | ASGA0003049 | 1 | 64036390 | *SRSF12, LOC100153533, GABRR1, LOC100156383, RRAGD, PM20D2, LOC100520705, RNGTT, LOC100522289, LOC100524470, LOC100739281, LOC100739399, LOC100739554, LOC100739627, UBE2J1* |
| DFI | ASGA0003051 | 1 | 64054552 | *SRSF12, LOC100153533, GABRR1, LOC100156383, RRAGD, PM20D2, LOC100520705, RNGTT, LOC100522289, LOC100523036, LOC100524470, LOC100739281, LOC100739399, LOC100739554, LOC100739627, UBE2J1* |
| DFI | ASGA0083328 | 1 | 64533206 | *GJA10, LOC100156383, RRAGD, LOC100522289, LOC100523036, LOC100523413, CASP8AP2, LOC100524470, MDN1, LOC100620522, LOC100627571, LOC100739554, LOC100739627, LOC100739733, UBE2J1* |
| DFI | H3GA0001815 | 1 | 49380268 | *LOC100154658, DCBLD1, ROS1, LOC100520342, GOPC, SLC35F1, LOC100739384* |
| DFI | H3GA0001822 | 1 | 64628578 | *GJA10, RRAGD, LOC100523036, LOC100523413, CASP8AP2, LOC100524470, MDN1, LOC100620522, LOC100627571, LOC100739554, LOC100739627, LOC100739733, UBE2J1* |
| DFI | MARC0076100 | 1 | 64510071 | *GJA10, GABRR1, LOC100156383, RRAGD, LOC100522289, LOC100523036, LOC100523413, CASP8AP2, LOC100524470, MDN1, LOC100620522, LOC100627571, LOC100739554, LOC100739627, LOC100739733, UBE2J1* |
| DFI | CAIL0000146 | 2 | 142136584 | *FRMD5, LOC100153375, SERINC4, SERF2, ELL3, PDIA3, MFAP1, LOC100517070, LOC100519285, LOC100523158, LOC100523528, LOC100625140, LOC100739268, LOC100739345* |
| DFI | ALGA0111383 | 9 | 118454584 | *SLC26A3, GPR22, NRCAM, LOC100622655, SLC26A4, LOC100623096, LOC100623191, LOC100623288, LOC100623385, LOC100623482, LOC100623759, LOC100736578, LOC100736621, LOC100736654, LAMB1, DLD* |
| DFI | ALGA0058575 | 10 | 44428078 | *LOC100517051, LOC100517599, LOC100517775, LOC100621185, LOC100621649, LOC100621751, BAMBI* |
| FPV | ALGA0102707 | NA | NA |  |
| FPV | ALGA01028861 | NA | NA |  |
| FPV | ALGA01188921 | NA | NA |  |
| FPV | ASGA01007851 | NA | NA |  |
| FPV | DRGA0017416 | NA | NA |  |
| FPV | M1GA00245242 | NA | NA |  |
| FPV | ALGA0042544 | 7 | 78311813 | *LOC100155683, LOC100158068, LOC100623004* |
| FPV | MARC0001935 | 7 | 77275077 | *NOVA1* |
| FPV | ASGA0048906 | 10 | 70074394 | *LOC100515404, LOC100515572, LOC100515751, LOC100620508, LOC100621009, LOC100621326, ITIH2, GATA3* |
| FPV | M1GA00143421 | 10 | 38849933 | *LOC100514001, LOC100737160* |
| FPV | MARC00914141 | 10 | 38641420 | *LOC100514001, ACO1, LOC100737160, DDX58* |
| FPV | ALGA00826662 | 14 | 139614808 | *C14H10orf84, RAB11FIP2, LOC100154968* |
| FPV | H3GA00383331 | 14 | 2744716 | *SYK, AUH, LOC100522346, LOC100739440, LOC780430* |
| FPV | ALGA0084813 | 15 | 37382667 | *DLGAP2, CLN8* |
| FPV | MARC0104064 | 15 | 36548921 | *LOC100521408, LOC100522166, PTPN4, LOC100523095, LOC100523470, LOC100623642, LOC100624144, LOC100624229* |
| FPV | ALGA0090475 | 16 | 42490292 | *LOC100523871, LOC100524052, LOC100524224, LOC100524405, LOC100524592, LOC100525133, LOC100738470* |
| FPV | ALGA00975731 | 18 | 13446026 | *LOC100521530, LOC100621475, PTF-BETA* |
| FPV | ASGA00793002 | 18 | 13082762 | *LOC100521167, LOC100521530, LOC100621475, PTF-BETA* |
| FPV | ASGA00793331 | 18 | 13503866 | *LOC100521530, LOC100621475* |
| FPV | DRGA00169471 | 18 | 13583864 | *LOC100621475* |
| FR | ASGA0089165 | NA | NA |  |
| FR | ASGA0007170 | 1 | 289023540 | *ASTN2* |
| FR | ASGA0007175 | 1 | 289350190 | *ASTN2, TLR4* |
| FR | H3GA0006163 | 2 | 18828505 | *CD82, SYT13, PRDM11, LOC100520600, LOC100520775* |
| FR | DRGA0006450 | 5 | 107536603 | *SYT1, LOC100736848* |
| FR | ALGA00493341 | 8 | 57187784 | *LOC100519499, LOC100519670, LOC100519853, LOC100520393, LOC100520576, REST, LOC100625602, LOC100625795, LOC100625851, LOC100626988, HOPX* |
| FR | DRGA00087791 | 8 | 57141466 | *LOC100519499, LOC100519670, LOC100519853, LOC100520393, LOC100520576, REST, LOC100625602, LOC100625795, LOC100625851, LOC100626988, HOPX* |
| FR | H3GA0025364 | 8 | 124894821 | *LOC100520756, LOC100521105, TET2, LOC100624822, LOC100625394, LOC100738766, LOC100738848* |
| FR | MARC0098171 | 8 | 124871992 | *LOC100520756, LOC100521105, TET2, LOC100624822, LOC100625394, LOC100738766, LOC100738848* |
| FR | ASGA00495811 | 11 | 6392619 | *LOC100156612, LOC100157816, LOC100517418, LOC100620545, MTUS2, LOC100738037, SLC7A1* |
| FR | DRGA0017669 | 16 | 76276873 | *NMUR2, LOC100624020* |
| NVD | MARC0030253 | NA | NA |  |
| NVD | H3GA0029068 | 10 | 5156226 | *LOC100512300, LOC100512672* |
| NVD | ALGA0065784 | 12 | 26506162 | *SGCA, SPATA20, MYST2, LOC100511101, EPN3, ACSF2, LOC100513042, CACNA1G, MYCBPAP, LRRC59, LOC100514487, LOC100514793, LOC100514982, LOC100515167, LOC100515340, LOC100515686, LOC100515855, PDK2, LOC100516335, LOC100516510, LOC100516875, ITGA3, LOC100517238, LOC100624069, LOC100625662, LOC100737233, LOC100737651, LOC100737860, LOC100738085, LOC100738213, LOC100738384, LOC100738426, LOC100738465, LOC100738512, COL1A1* |
| NVD | ALGA0066091 | 12 | 34781411 | *MRPS23, VEZF1, CUEDC1, LOC100737199, LOC100737226, LOC100737430, LOC100737474, LOC100737515, SRSF1* |
| NVD | ASGA0054137 | 12 | 33907320 | *DGKE, LOC100524708, LOC100525005, ANKFN1, LOC100525534, LOC100525713, SCPEP1, LOC100526243, LOC100622285, LOC100737114* |
| NVD | ASGA0054177 | 12 | 34360905 | *DGKE, LOC100524708, LOC100525005, LOC100525534, LOC100525713, SCPEP1, LOC100526243, LOC100622285, LOC100737114, LOC100737199, LOC100737226* |
| NVD | ASGA0054288 | 12 | 34781411 | *MRPS23, VEZF1, CUEDC1, LOC100737199, LOC100737226, LOC100737430, LOC100737474, LOC100737515, SRSF1* |
| NVD | ASGA0054337 | 12 | 36387623 | *CLTC, MIR142, LOC100513817, EPX, MKS1, LOC100514728, BZRAP1, LOC100515097, LOC100515407, LOC100515575, LOC100516395,LOC100516575, LOC100516756, LOC100516933, MPO, LOC100517305, LOC100517493, LOC100517675, PPM1E, TRIM37, LOC100519272, LOC100524105, LOC100524457, TEX14, LOC100738790, LOC100738832, LOC100738997* |
| NVD | ASGA0084073 | 12 | 34087275 | *DGKE, LOC100524708, LOC100525005, ANKFN1, LOC100525534, LOC100525713, SCPEP1, LOC100526243, LOC100622285, LOC100737114, LOC100737199* |
| NVD | H3GA0034044 | 12 | 32074865 | *TOM1L1, LOC100522894, LOC100736688, LOC100736723, LOC100739396, LOC100739466, LOC100739503, STXBP4, LOC100739655, MMD* |
| NVD | M1GA0016584 | 12 | 34552177 | *DGKE, MRPS23, CUEDC1, LOC100524708, LOC100525713, LOC100622285, LOC100737114, LOC100737199, LOC100737226* |
| NVD | MARC0011225 | 12 | 32136826 | *TOM1L1, LOC100522894, LOC100736688, LOC100736723, LOC100739396, LOC100739466, LOC100739503, STXBP4, LOC100739655, MMD* |
| NVD | MARC0045984 | 12 | 34151283 | *DGKE, LOC100524708, LOC100525005, LOC100525534, LOC100525713, SCPEP1, LOC100526243, LOC100622285, LOC100737114, LOC100737199, LOC100737226* |
| NVD | MARC0070458 | 12 | 34719298 | *MRPS23, VEZF1, CUEDC1, LOC100737199, LOC100737226, LOC100737430, LOC100737474* |
| NVD | MARC0072638 | 12 | 34381325 | *DGKE, LOC100524708, LOC100525005, LOC100525534, LOC100525713, SCPEP1, LOC100526243, LOC100622285, LOC100737114, LOC100737199, LOC100737226* |
| NVD | MARC0097496 | 12 | 39543788 | *LOC100511040, CA4, USP32, LOC100512308, GGNBP2, DHRS11, MRM1, LOC100514070, APPBP2, LOC100620905, LOC100736689, LOC100736724, LOC100736935, LOC100737200, LOC100737416* |
| NVD | ASGA0066557 | 14 | 134702823 | *TCF7L2, HABP2, LOC100516762, LOC100516880, NRAP* |
| NVD | H3GA0038333 | 14 | 2865914 | *SYK, NFIL3, AUH, LOC100522346, LOC100739440, LOC780430* |
| NVD | MARC0080034 | 14 | 134634209 | *TCF7L2, HABP2, LOC100516762, LOC100516880, NRAP* |
| NVD | ALGA0092509 | 17 | 509344 | *CLDN23, LOC100524773, LOC100524963, LOC100525134, LONRF1, LOC100623742, LOC100623836* |
| NVD | ASGA0079300 | 18 | 26316841 | *LOC100510940, FEZF1, PTPRZ1, LOC100513769, AASS, CADPS2* |
| NVD | DRGA0016947 | 18 | 26825286 | *FEZF1, PTPRZ1, LOC100511484, LOC100511668, C18H7orf58, AASS, LOC100514140, LOC100514329, LOC100514502, CADPS2* |
| TPD | ASGA0095686 | NA | NA |  |
| TPD | ASGA0105377 | NA | NA |  |
| TPD | M1GA0026294 | NA | NA |  |
| TPD | INRA0020528 | 5 | 102326523 | *LOC100627747, LRRIQ1, LOC100628231, LOC100739274, LOC100739317* |
| TPD | MARC0085057 | 5 | 101511939 | *ALX1, LOC100526053, LOC100526222, LOC100627747, LRRIQ1, NTS, LOC100739274* |
| TPD | ALGA0049421 | 8 | 129335905 | *H2AFZ, LOC100511703, DNAJB14, LOC100525593, LOC100525762, LOC100525936, LOC100526122, LOC100620154, LOC100620684, LOC100620774, LOC100628129, PPP3CA* |
| TPD | ASGA0039757 | 8 | 128703259 | *LOC100511337, LOC100511703, LOC100525061, LOC100628129, PPP3CA* |
| TPD | ASGA0039827 | 8 | 130796392 | *EIF4E, ADH4, ADH5, LOC100513747, LOC100514117, LOC100514305, LOC100622240, RAP1GDS1* |
| TPD | H3GA0025421 | 8 | 129600171 | *H2AFZ, LOC100511703, DNAJB14, RG9MTD2, LOC100525593, LOC100525762, LOC100525936, LOC100526122, LOC100620154, LOC100620684, LOC100620774, LOC100628129, LOC100737055, LOC100737351, MTTP* |
| TPD | ALGA0060579 | 11 | 6845024 | *LOC100156612, LOC100517418, USPL1, ALOX5AP, HMGB1, SLC7A1* |
| TPD | ALGA0060596 | 11 | 7421327 | *HSPH1, LOC100153765, LOC100516873, LOC100517236, USPL1, ALOX5AP, HMGB1* |
| TPD | ALGA0060626 | 11 | 6443449 | *LOC100156612, LOC100157816, LOC100517418, LOC100620545, LOC100738037, SLC7A1* |
| TPD | ASGA0049581 | 11 | 6392619 | *LOC100156612, LOC100157816, LOC100517418, LOC100620545, MTUS2, LOC100738037, SLC7A1* |
| TPD | ASGA0049606 | 11 | 8523653 | *LOC100153603, LOC100154012, LOC100154396, LOC100155774, FRY, LOC100621650* |
| TPD | ASGA0049612 | 11 | 8505201 | *LOC100153603, LOC100154012, LOC100155774, FRY, LOC100621650* |
| TPD | M1GA0014839 | 11 | 6640240 | *LOC100156612, LOC100157816, LOC100517418, LOC100620545, LOC100738037, HMGB1, SLC7A1* |
| TPD | ASGA0068413 | 15 | 7579316 | *LOC100156520, LOC100622892, LOC100623074, LOC100739608, LOC100739669* |
| TPV | ALGA0035106 | 6 | 30009402 | *SALL1, LOC100624570* |
| TPV | ALGA0103136 | 6 | 30028044 | *SALL1, LOC100624570* |
| TPV | MARC0053050 | 6 | 29469612 | *LOC100519545, TOX3* |
| TPV | MARC0091414 | 10 | 69589046 | *LOC100515404, LOC100515572, LOC100620508, ITIH2, GATA3* |
| TPV | ALGA0102886 | 12 | 59982065 | *LOC100520637, ELAC2, LOC100520977, LOC100737652, MYOCD* |
| TPV | ALGA0118892 | 12 | 60027710 | *LOC100520637, ELAC2, LOC100520977, LOC100737652, MYOCD* |
| TPV | ASGA0100785 | 12 | 59966291 | *LOC100520637, ELAC2, LOC100520977, LOC100737652, MYOCD* |
| TPV | M1GA00245241 | 12 | 59746968 | *ZNF18, DNAH9, LOC100520100, LOC100520637, ELAC2, LOC100520977, LOC100737652, MYOCD* |
| TPV | H3GA0054084 | 15 | 35839572 | *SCTR, LOC100521408, PTPN18, LOC100521754, LOC100522166, LOC100522523, PTPN4, LOC100523095, LOC100623642, LOC100736657, LOC100737074* |
| TPV | ALGA0095059 | 17 | 45969331 | *LOC100152865, RBL1, RPN2, SAMHD1, SRC, TTI1, LOC100155728, LOC100156518, C20orf118, GHRH, LOC100512993, LOC100513892, LOC100514259, LOC100515108, LOC100627266, LOC100736843, LOC100736939, LOC100737342, NNAT* |
| TPV | DRGA00169471 | 18 | 13583864 | *LOC100621475* |

^1^: DFI: total daily feed intake, FPV: mean feed intake per visit, FR: mean feed intake rate, NVD: number of visits to the feeder per day, TPD: total time spent at feeder per day, TPV: time spent to eat per visit

^2^: Candidate genes shown in EntrezID
