# Supplementary material for: Genome-Wide Association Study Reveals Genetic Architecture of Eating Behavior in Pigs and Its Implications for Humans Obesity by Comparative Mapping
Source: PLoS One. 2013 Aug 19;8(8):e71509. doi: 10.1371/journal.pone.0071509 (PMC3747221; doi:10.1371/journal.pone.0071509)
Supplement: Table S4 — Haplotypes and their frequencies in the candidate region for number of visits to feeder per day on chromosome 12. (DOC) [file pone.0071509.s008.doc]

| LOCUS | HAPLOTYPE^1^ | F^2^ | R^2^ | SNPS |
| --- | --- | --- | --- | --- |
| BLOCK1 | 11211 | 0.025 | 0.06% | ALGA0065979|ALGA0065989|ALGA0066000|H3GA0034093|ALGA0066020 |
| BLOCK1 | 22211 | 0.043 | 0.08% | ALGA0065979|ALGA0065989|ALGA0066000|H3GA0034093|ALGA0066020 |
| BLOCK1 | 12211 | 0.121 | 0.06% | ALGA0065979|ALGA0065989|ALGA0066000|H3GA0034093|ALGA0066020 |
| BLOCK1 | 21222 | 0.020 | 0.53% | ALGA0065979|ALGA0065989|ALGA0066000|H3GA0034093|ALGA0066020 |
| BLOCK1 | 11212 | 0.078 | 0.00% | ALGA0065979|ALGA0065989|ALGA0066000|H3GA0034093|ALGA0066020 |
| BLOCK1 | 22212 | 0.130 | 0.01% | ALGA0065979|ALGA0065989|ALGA0066000|H3GA0034093|ALGA0066020 |
| BLOCK1 | 12212 | 0.573 | 0.24% | ALGA0065979|ALGA0065989|ALGA0066000|H3GA0034093|ALGA0066020 |
| BLOCK2 | 11 | 0.245 | 0.04% | ALGA0066091|MARC0037857 |
| BLOCK2 | 21 | 0.248 | 0.03% | ALGA0066091|MARC0037857 |
| BLOCK2 | 12 | 0.248 | 0.08% | ALGA0066091|MARC0037857 |
| BLOCK2 | 22 | 0.259 | 0.00% | ALGA0066091|MARC0037857 |

**Table S4. Haplotypes, their frequencies in the candidate region for number of visits to feeder per day on chromosome 12**

^1^ 1 is minor alleles and 2 is major allele

^2^ Percentage of deregressed EBV of number of visits to feeder per day explained by markers based on association tests
